# Influence of the Nature of Group 15 Element on [Au^I^]–C≡E/Azide 1,3-Dipolar Cycloaddition Reaction

**DOI:** 10.1021/acs.inorgchem.5c00110

**Published:** 2025-03-12

**Authors:** Daniel González-Pinardo, Israel Fernández

**Affiliations:** Departamento de Química Orgánica and Centro de Innovación en Química Avanzada (ORFEO-CINQA), Facultad de Ciencias Químicas, 16734 Universidad Complutense de Madrid, 28040 Madrid, Spain

## Abstract

The impact of the nature of the Group 15 element on both the bonding situation and the reactivity of gold­(I)-C ≡ E (E = N to Bi) complexes has been studied quantum chemically within the density theory functional framework. For this purpose, the 1,3-dipolar cycloaddition reaction involving *t*BuN_3_ as dipole has been selected and its main features, including the regioselectivity of the transformation and the in-plane aromaticity of the corresponding transition structures, have been investigated. It is found that the reactivity of the complexes is increased as one moves down Group 15 (N ≪ *P* < As < Sb < Bi). This reactivity trend has been rationalized by using the combined activation strain model and energy decomposition analysis methods, which indicate that the process is mainly dominated by the strain energy required by the reactants to reach the corresponding transition state geometries.

## Graphical abstract

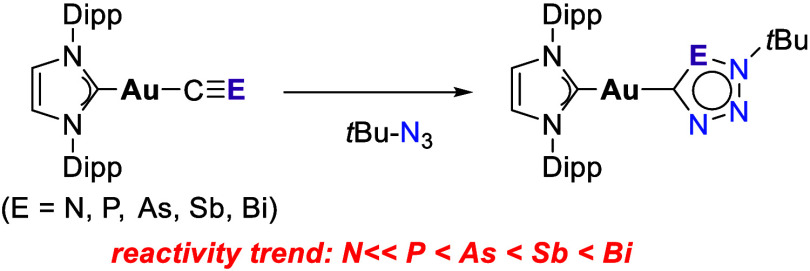

## Introduction

1,3-Dipolar cycloaddition is a powerful and versatile reaction in organic and organometallic chemistry, able to construct five-membered heterocycles with high atom economy and regioselectivity.^1,2^ This transformation typically involves the reaction between a 1,3-dipole (molecules or ions with delocalized charges acting as a 4π system) and a dipolarophile (usually a 2π unsaturated species) in a [3 + 2] cycloaddition leading to the heterocyclic cycloadduct.^3^ Although initially introduced by R. Husigen in 1960,^4^ this process has experienced a new renaissance since the introduction of the so-called “click chemistry” by B. Sharpless^5^ and its countless applications, particularly in bioorthogonal chemistry.^6^

Although alkynes and alkenes are arguably the most popular dipolarophiles used in 1,3-dipolar cycloadditions, related systems based on the main-group elements have emerged for the preparation of novel heterocycles. For instance, phosphalkynes (R-C≡P)^7^ or arsaalkynes (R-C≡As)^8^ can undergo 1,3-dipolar cycloadditions with organic azides to afford triazaphospholes and triazaarsoles, respectively, compounds with potential applications as tunable ligand scaffolds and as π-conjugated luminescent species in materials science.^9^ However, due to a less efficient overlap of the p orbitals, these Group 15 alkynes are typically kinetically unstable (i.e., leading to oligomerization reactions) which severely hampers their application as dipolarophiles.^10^ Indeed, as a consequence of a dearth of isolable phosphaalkynes, for instance, only triazaphospholes with bulky alkyl, aryl, or silyl substituents have been produced using [3 + 2] cycloaddition reactions.^9b,10^ Fortunately, recent reports by Müller, Jones, and co-workers^11^ and by Goicoechea et al.^12^ indicate that the C≡P moiety, in particular, can be stabilized upon binding to a transition metal or main-group fragment, therefore forming a stable cyaphide complex which readily undergoes a 1,3-dipolar with azides to produce the corresponding organometallic triazaphosphole (Scheme 1). The formed cycloadduct can be further transformed into protio- and iodo-triazaphospholes,^13^ and in the case of gold derivatives, this reaction can be used as a stoppering strategy for the preparation of interlocked molecules,^14^ which nicely illustrates the usefulness of this cyaphide stabilization strategy.^15^

**Scheme 1:**
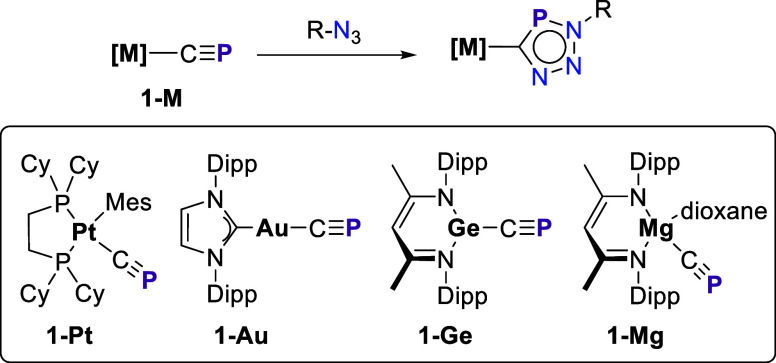
1,3-Dipolar Cycloaddition Reactions Involving Cyaphide Complexes Described in the Literature

Intrigued by this transformation, we have recently explored the impact of the transition metal moiety on the reactivity of different cyaphide complexes in their 1,3-dipolar cycloadditions with azides.^16^ It was found that although gold­(I) and platinum­(II)-cyaphides (**1-Pt** and **1-Au**, Scheme 1) exhibit a barrier comparable to that of the parent organic reaction involving *t*BuC≡P, the analogous cycloaddition reactions involving germanium and, particularly, magnesium cyaphide complexes (**1-Ge** and **1-Mg**) proceed with lower barriers. The factors behind this reactivity trend were analyzed in a quantitative manner with the help of the activation strain model (ASM)^17^ of reactivity combined with the energy decomposition analysis (EDA)^18^ method, an approach that has greatly contributed to our understanding of different reactions in organic, organometallic, and main-group chemistry,^19^ and particularly, related cycloaddition reactions.^20^ Despite that, the influence of the nature of the Group 15 element on this transformation is essentially unknown. This encouraged us to perform a thorough computational study to understand the role of the C≡E ligand (E = N to Bi) in gold­(I) complexes, analogous to the [Au^I^]-cyaphide complex **1-Au** described by Beer, Goicoechea, and co-workers,^12,13^ in their 1,3-dipolar cycloaddition with *t*BuN_3_ (Scheme 2). Issues such as the bonding situation of the initial complexes as well as the regioselectivity of the transformation, aromaticity of the corresponding transition states, and reactivity trends are analyzed in detail.

**Scheme 2:**
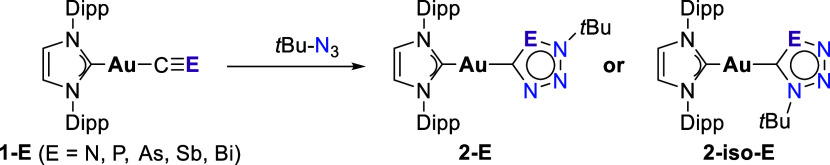
1,3-Dipolar Cycloaddition Reactions Investigated in This Study

### Computational Details

Geometry optimizations of the molecules were performed without symmetry constraints using the Gaussian16 (RevB.01) suite of programs.^21^ Calculations were conducted at the M06-2X^22^/def2-SVP^23^ level, incorporating solvent effects (solvent = toluene) via the Polarization Continuum Model (PCM) method.^24^ All species were characterized by frequency calculations: reactants and adducts exhibited positive definite Hessian matrices, while transition states showed a single negative eigenvalue in their diagonalized force constant matrices. The corresponding eigenvectors were verified to represent the motion along the considered reaction coordinate using the intrinsic reaction coordinate (IRC) method.^25^ Energy refinements were performed through single-point calculations at the same DFT level but with the much larger triple-ζ basis set def2-TZVPP.^23^ The computed thermochemistry data were corrected following Grimme’s quasi-harmonic (QHA) model for entropy^26^ with a frequency cutoff value of 100.0 cm^–1^ and corrected to a 1 M concentration using the GoodVibes^27^ program at 298.15 K. This level is denoted as PCM­(toluene)-M06-2X/def2-TZVPP//PCM­(toluene)-M06-2X/def2-SVP.

The aromaticity of the corresponding transition states was evaluated by calculating the nuclear independent chemical shift (NICS)^28^ values using the gauge invariant atomic orbital (GIAO) method^29^ at the B3LYP/def2-SVP//PCM­(toluene)-M06-2X/def2-SVP level. Ring currents were computed using the anisotropy of the induced current density (ACID) method.^30^

#### Activation Strain Model of Reactivity and Energy Decomposition Analysis

In the ASM method,^17^ also known as the distortion/interaction model,^17c^ the potential energy surface Δ*E*(ζ) can be divided into two contributions: the strain Δ*E*_strain_(ζ) associated with the deformation (or distortion) required by the reactants during the reaction and the interaction Δ*E*_int_(ζ) between these increasingly distorted reactants along the reaction coordinate, ζ:

$$\Delta E\left(\zeta \right)=\Delta E_{\mathrm{strain}}\left(\zeta \right)+\Delta E_{\mathrm{int}}\left(\zeta \right)$$

Within the EDA method,^18^ the Δ*E*_int_(ζ) term can be further decomposed into the following contributions:

$$\Delta E_{\mathrm{int}}\left(\zeta \right)=\Delta V_{\mathrm{elstat}}\left(\zeta \right)+\Delta E_{\mathrm{Pauli}}\left(\zeta \right)+\Delta E_{\mathrm{orb}}\left(\zeta \right)$$

The term Δ*V*_elstat_ represents the classical electrostatic interaction between the unperturbed charge distributions of the distorted reactants and is typically attractive. The Pauli repulsion Δ*E*_Pauli_ accounts for destabilizing interactions between occupied orbitals and is responsible for any steric repulsion. The orbital interaction Δ*E*_orb_ encompasses bond pair formation, charge transfer (interaction between occupied and unoccupied orbitals on both reactants, including HOMO–LUMO interactions), and polarization (empty-occupied orbital mixing on one fragment due to the presence of another fragment). Moreover, the natural orbital for chemical valence (NOCV)^31^ extension of the EDA method can be also applied to further partition the Δ*E*_orb_ term. The EDA-NOCV approach provides pairwise energy contributions for each pair of interacting orbitals to the total bond energy.

The ADF^32^ program package was used for EDA calculations using the optimized PCM­(toluene)-M06-2X/def2-SVP geometries at the same M06-2X level in combination with a triple-ζ-quality basis set using uncontracted Slater-type orbitals (STOs) augmented by two sets of polarization functions with a frozen-core approximation for the core electrons.^33^ Auxiliary sets of s, p, d, f, and g STOs were used to fit the molecular densities and to represent the Coulomb and exchange potentials accurately in each SCF cycle.^34^ Scalar relativistic effects were included by using the zeroth-order regular approximation (ZORA).^35^ This computational level is denoted as ZORA-M06-2X/TZ2P//PCM­(toluene)-M06-2X/def2-SVP.

## Results and Discussion

Before exploring the influence of the E atom on the reactivity, we first studied the bonding situation in the starting [Au^I^]-C≡E complexes **1-E**. To this end, we first applied the energy decomposition analysis (EDA) method.^18^ As reported previously by Goicoechea^36^ and more recently also by us,^16^ the fragmentation scheme using charged [Au^I^]^+^/[C≡E]^−^ fragments, i.e., leading to a donor–acceptor interaction, better describes the bonding situation of these complexes. Using this partitioning, we found that the interaction energy between the [Au^I^]^+^ and [C≡E]^−^ fragments is rather similar (Δ*E*_int_ ca. −161 to −165 kcal/mol) in all complexes with the exception of the cyanide (E = N) complex, which exhibits the weakest interaction in the entire series (Table 1).^37^ This correlates with the computed Au–C­(E) distances which are rather similar for the heavier Group 15 systems (E = P to Bi, Au–C ≈ 1.990 Å, average value), whereas a longer distance was computed for the cyanide complex (E = N, Au–C = 2.020 Å). Similarly, the cyanide complex exhibits the lowest Pauli repulsion (Δ*E*_Pauli_) deriving from a smaller orbital overlap (*S*) between the cyanide lone pair and the occupied d_z2_ atomic orbital of the transition metal (for instance, a value of 0.049 was computed for **1-CN**, whereas a larger value of 0.071 was found for the analogous cyaphide complex). As expected, the electrostatic attractions are much stronger than the orbital interactions in all complexes, which mainly derives from the charged nature of the selected fragments.^38^ Despite that, the cyanide complex also exhibits the weaker Δ*V*_elstat_ term as a consequence of the higher electronegativity of the nitrogen atom which polarizes the C≡N bond toward the nitrogen end. This is supported by the computed NBO charges^39^ at the carbon atom of the C≡E ligand which are again rather similar for the heavier systems (ranging from −0.96 to −1.09e), while a much less negative value was computed for the cyanide counterpart (−0.12e).

**Table 1:** EDA Values (in kcal/mol) of the [Au]-C ≡ E Complexes (E = N to Bi)^a^

complex **1-E**	Δ*E*_int_	Δ*E*_Pauli_	Δ*V*_elstat_	Δ*E*_orb_	Δ*E*(ρ1)	Δ*E*(ρ2)	Δ*E*(ρ3)
[Au]-CN	–156.7	159.1	–248.2	–67.6	–46.4	–7.4	–7.2
[Au]-CP	–165.2	205.3	–293.6	–76.9	–46.0	–9.6	–10.0
[Au]-CAs	–163.2	204.6	–288.5	–79.4	–45.6	–10.7	–11.2
[Au]-CSb	–160.7	207.4	–285.5	–82.6	–46.6	–12.0	–12.7
[Au]-CBi	–162.5	205.2	–281.5	–86.1	–49.3	–11.2	–12.2

a All data were computed at the ZORA-M06-2X/TZ2P//PCM­(toluene)-M06-2X/def2-SVP level.

The computed orbital interactions between the charged fragments also follow a trend similar to that of Δ*E*_int_ or Δ*V*_elstat_, in the sense that the cyanide complex exhibits the less stabilizing Δ*E*_orb_ term. Despite that, there exists a clear increase in the Δ*E*_orb_ strength when moving down in Group 15 from P to Bi. To understand this trend, we applied the natural orbital for chemical valence (NOCV)^31^ extension of the EDA method, which allows us to quantify the main orbital interactions contributing to the total orbital interaction term. According to the NOCV method, three main orbital interactions dominate the Δ*E*_orb_ term, namely, the σ-donation from the lone pair at the carbon atom of the C≡E ligand to an empty (mainly s orbital) of the transition metal (denoted as ρ1 in Figure 1) and two π-backdonations from a doubly occupied d atomic orbital of the transition metal to the vacant, degenerate π*-molecular orbitals of the C≡E ligand (denoted as ρ2 and ρ3, respectively). From the data in Table 1, it is clear that in all cases, the σ-donation is comparatively much stronger than the π-backdonations. Interestingly, according to the associated stabilization energies (Δ*E*(ρ)), while the σ-donation (ρ1) is quite similar in all cases, the π-backdonations steadily increase from N to Sb ≈ Bi. Therefore, this indicates that the C≡E ligand exhibits a similar σ-donation ability regardless of the nature of the E element (except for bismuth which shows a slightly stronger Δ*E*(ρ1) value), while its π-acceptor ability increases when going down in the group. This enhanced π-acceptor ability when going from N to Bi is therefore behind the computed trend in the total orbital interactions.

**Figure 1:**
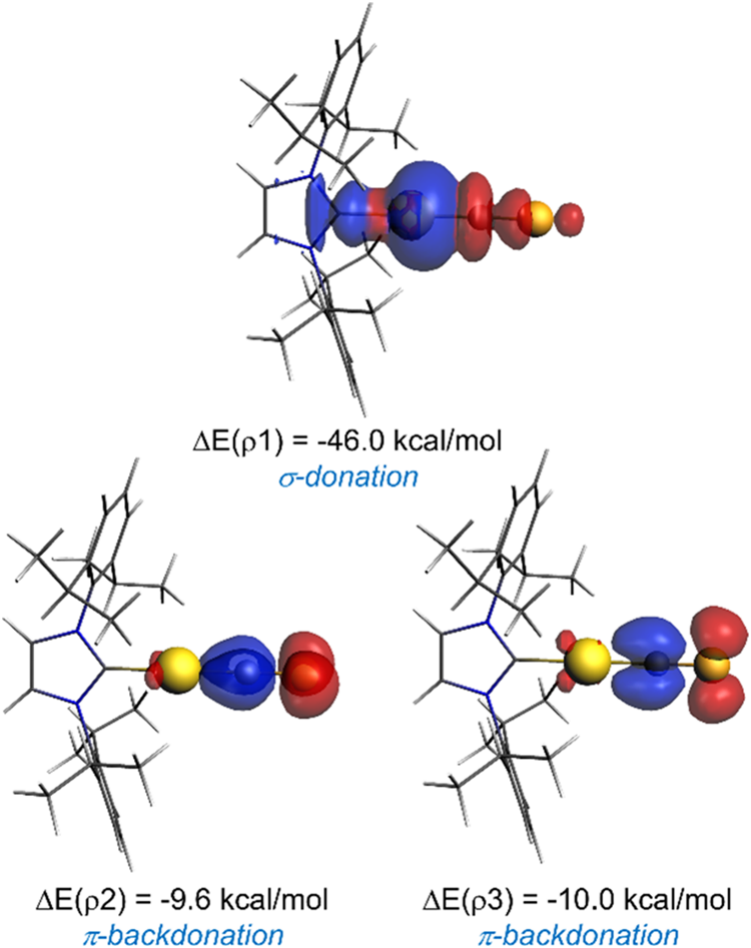
Plots of the main NOCV deformation densities ρ (isosurface value of 0.001 au) and associated energies Δ*E*(ρ) in **1-E** (E = N to Bi). The electronic charge flows in the red → blue direction. All data were computed at the ZORA-M06-2X/TZ2P//PCM­(toluene)-M06-2X/def2-SVP level.

Once the bonding situation in the initial gold­(I)-complexes **1-E** has been studied, we then explored their reactivity in the [3 + 2] cycloaddition with *t*BuN_3_ as a dipole. Figure 2 depicts the corresponding reaction profiles leading to the two possible regioisomers **2-E** and **2-iso-E**. Our calculations indicate that in all cases, the 1,3-dipolar cycloaddition reaction is concerted involving a five-membered transition state (**TS** or **TS-iso**) which directly produces the corresponding cycloadduct (**2-E** or **2-iso-E**). Data in Figure 2 indicate that although the process is strongly exergonic for the heavier (E = P to Bi) complexes, the analogous reactions involving the cyanide complex are slightly endergonic, which suggests that the former reactions are irreversible while the latter would be reversible. In addition, the activation barriers gradually decrease as one moves down Group 15. Despite that, although the cycloadditions are feasible for the heavier complexes (Δ*G* ^‡^ ranging from 26.3 kcal/mol for E = P to 16.7 kcal/mol for E = Bi), the cyanide complex exhibits a prohibitive barrier of ca. 50 kcal/mol. This can be initially attributed to the relative strength of the reactive C≡E bond in the initial complexes **1-E** as confirmed by the corresponding Wiberg bond orders (N = 2.91 > P = 2.87 > As = 2.82 > Sb = 2.74 ≈ Bi = 2.74), which, as commented above, results from a less efficient overlap of the p orbitals involving the heavier (than nitrogen) elements. Therefore, our calculations predict that the so-far not described heavy systems **1-As**, **1-Sb**, and **1-Bi** are even more reactive than the reported **1-P**,^13^ and can be then considered as possible experimental targets for the preparation of heavy triazaphosphole analogues. Interestingly, a rather similar reactivity trend is found in the analogous [3 + 2]-cycloaddition reactions involving the nonmetallic systems *t*Bu-C≡E: Δ*G* ^‡^ = 48.6 (E = N) > 25.7 (E = P) > 22.6 (E = As) > 16.9 (E = Sb) > 15.6 (E = Bi) kcal/mol (data for the preferred cycloadduct), thus confirming the significant influence of the nature of the Group 15 element on the transformation.

**Figure 2:**
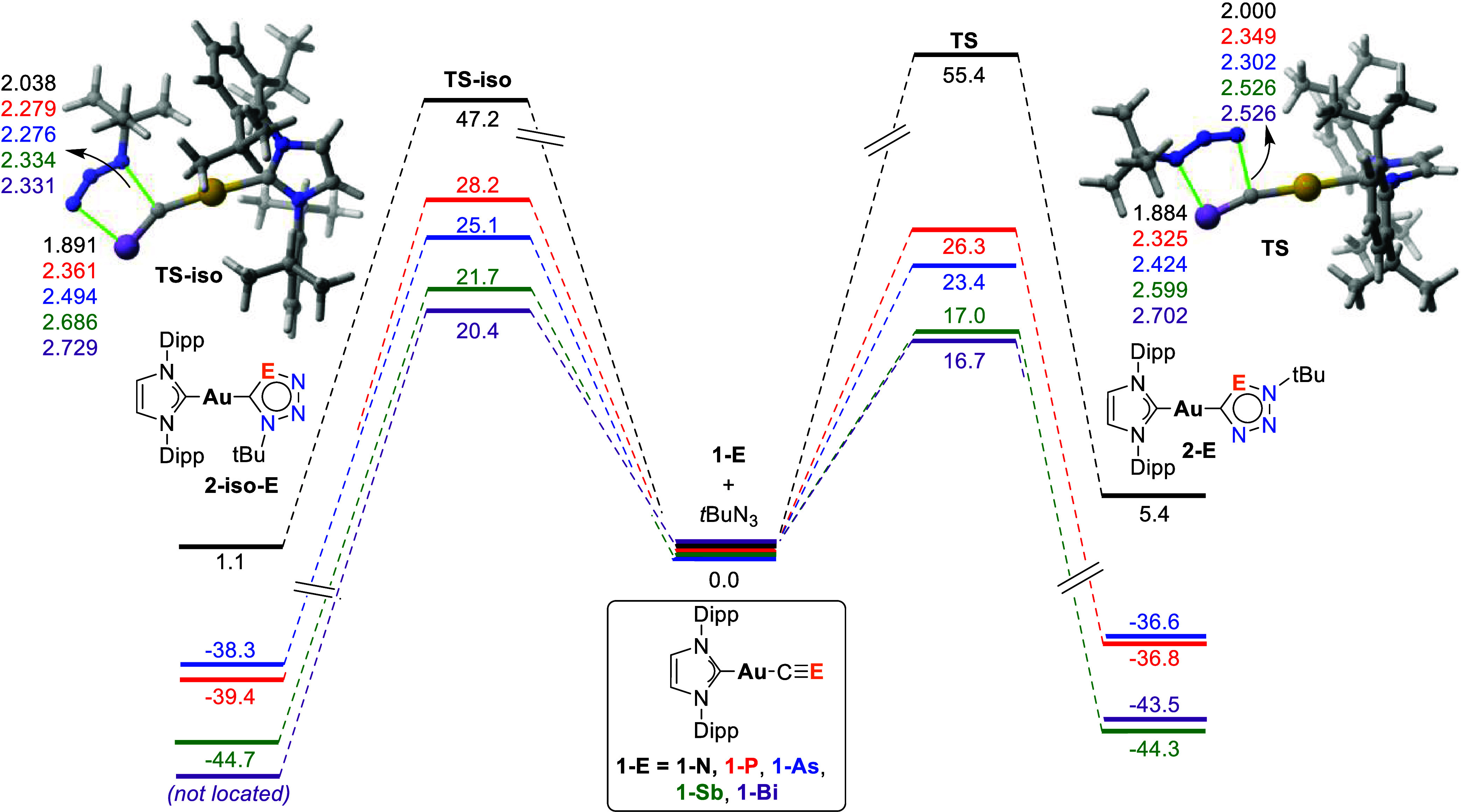
Computed reaction profiles for the 1,3-dipolar cycloaddition reactions between *t*BuN_3_ and complexes **1-E**. Relative free energies (Δ*G*, at 298 K) and bond lengths are given in kcal/mol and angstroms, respectively. All data were computed at the PCM­(toluene)-M06-2X/def2-TZVPP//PCM­(toluene)-M06-2X/def2-SVP level.

Data in Figure 2 also indicate that the cycloaddition is regioselective toward the formation of the cycloadducts **2-E** in view of the lower barriers computed for the processes involving **TS** in comparison to the analogous cycloadditions involving **TS-iso**. This is also consistent with the regioselectivity observed experimentally in the 1,3-dipolar cycloaddition reactions involving **1-P**.^13^ Once again, the cyanide complex exhibits a different behavior than its heavier counterparts in view of the lower (although unfeasible) barrier computed for the formation of the **2-iso-N** regioisomer as compared to that involved in the formation of **2-N**. This can be rationalized by the different polarization of the C≡E bond in the initial gold­(I)-complexes induced by the Group 15 element. As commented above, the much higher electronegativity of the nitrogen atom polarizes the C≡E bond toward the nitrogen end, whereas the situation is the opposite in their heavier analogues; i.e., the carbon atom in **1-E** shows a charge accumulation for E = P to Bi (ranging from −0.96 to −1.09e), while in **1-N**, the negative charge is mainly concentrated at the nitrogen atom (−0.50e, see corresponding electrostatic potential maps in Figure 3). As the azide dipole also exhibits a charge accumulation at the substituted nitrogen atom (−0.41e, Figure 3), the formation of the **2-E** regioisomer is favored for E = P to Bi while the opposite (i.e., formation of **2-iso-E**) is expected for E = N, as confirmed by the computed profiles in Figure 2.

**Figure 3:**
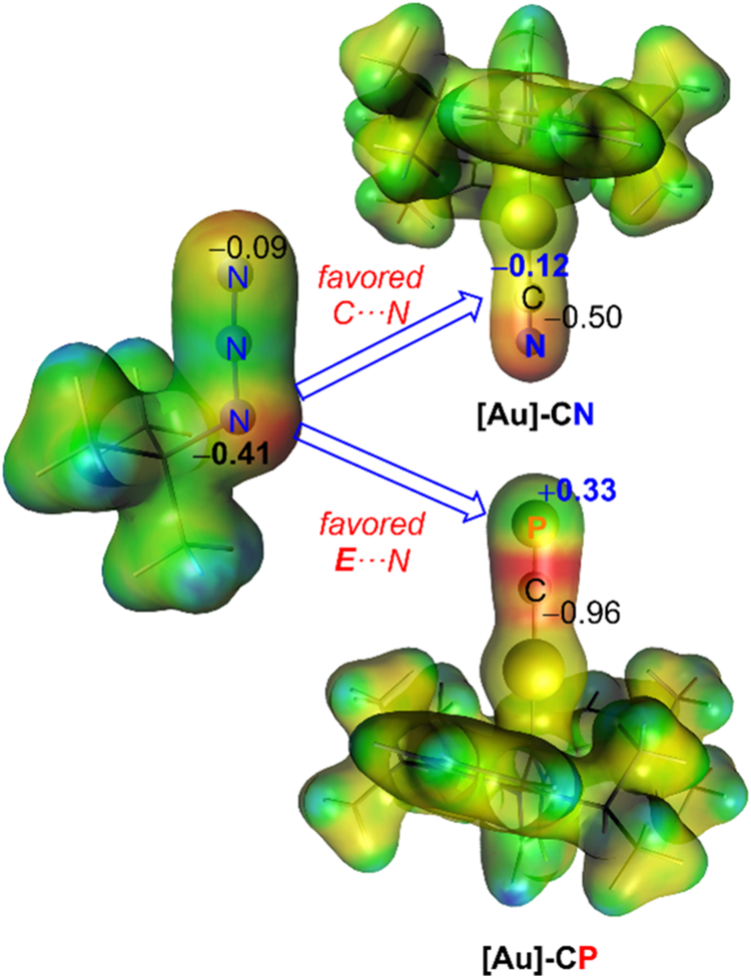
Electrostatic potential maps showing the preferred interaction between *t*BuN_3_ and **1-N** (top) and **1-P** (bottom) as representative examples of the heavier analogues. Values refer to the computed NBO charges of the atoms involved in the transformation.

Similar to other pericyclic reactions,^40,41^ the transition state involved in these particular [3 + 2]-cycloaddition reactions can be considered as aromatic species as they involve the delocalization of six electrons in a cyclic circuit.^42^ To assess the possible aromatic nature of these saddle points, we first computed the nuclear independent chemical shift (NICS)^28^ values at the (3,+1) ring critical point (RCP)^43^ of the corresponding five-membered cycles. In all cases, highly negative NICS­(3,+1) values (steadily decreasing from −25.0 ppm for E = N to −19.2 ppm for E = Bi) were found, which confirms the aromatic nature of these species. Moreover, the variation of the NICS values along the *z*-axis, perpendicular to the molecular plane, consistently exhibits a bell-shaped plot with a maximum value at *z* = 0 Å (i.e., at the RCP, Figure 4a). This pattern indicates that these transition states feature the so-called *in plane* aromaticity.^40,42^ This behavior derives from the delocalization of the six electrons within the molecular plane in the concerted cycloaddition, which leads to significant diamagnetic shielding at the RCP. This delocalization is also confirmed by using the anisotropy of the induced current density (AICD)^30^ method, which clearly confirms the occurrence of a diatropic (i.e., clockwise vectors)-induced current within the closed circuit (see Figure 4b for **TS-N** as a representative example; see Figure S1 in the Supporting Information for the corresponding AICD plots of the remaining transition states).

**Figure 4:**
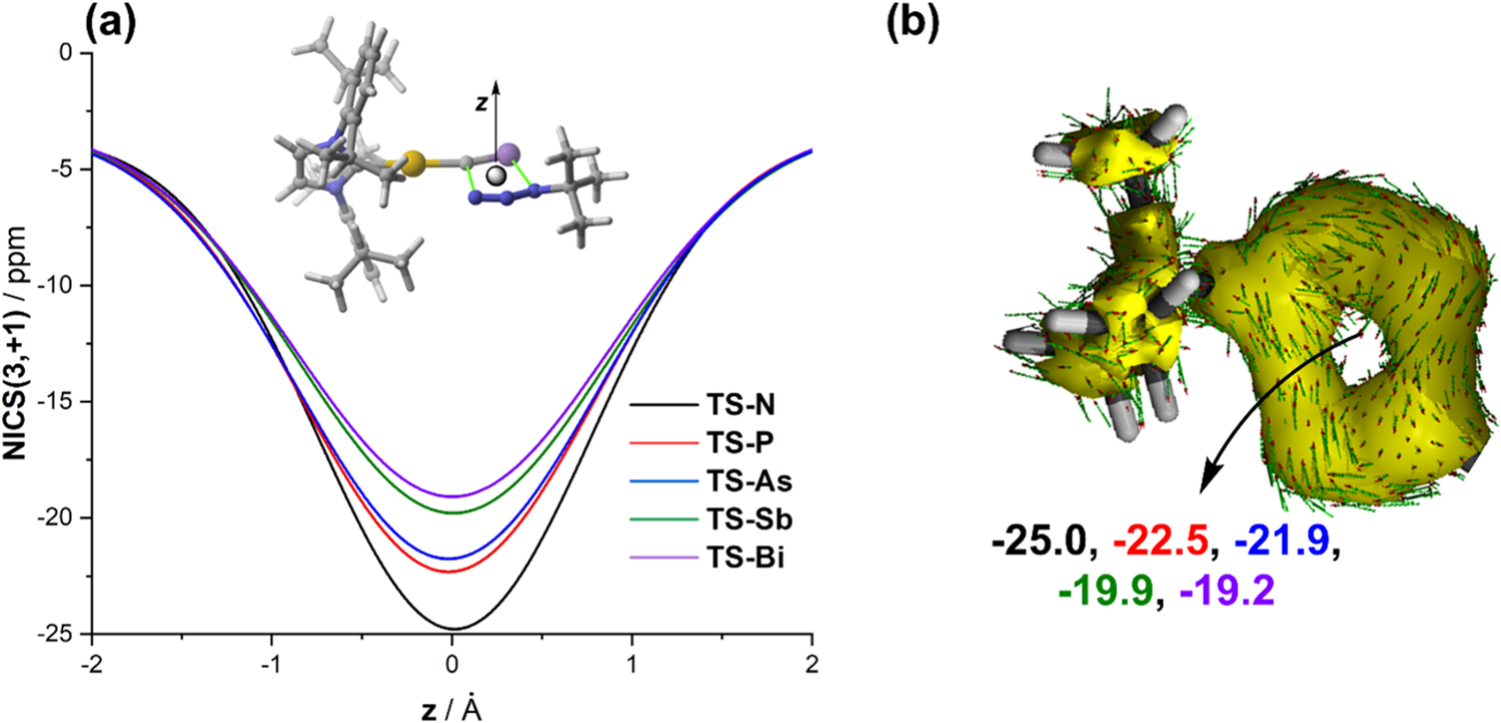
(a) Variation of the NICS­(3,+1) values along the *z*-axis perpendicular to the molecular plane of the considered transition states. (b) AICD plot (isosurface value of 0.03 au) of **TS-N**. The values indicate the computed NICS­(3,+1) values, in ppm, for **TS-N** (black), **TS-P** (red), **TS-As** (blue), **TS-Sb** (green), and **TS-Bi** (magenta).

The results above suggest that the aromaticity strength of the transition states (according to the computed NICS­(3,+1) values) does not control the different reactivities of the [Au^I^]-C≡E complexes. Thus, in order to understand the factors governing the computed reactivity trend (N ≪ *P* < As < Sb ≈ Bi), we then applied the activation strain model (ASM)^17^ of reactivity. Figure 5 shows the corresponding activation strain diagrams (ASDs) for the representative 1,3-dipolar cycloadditions involving **1-N** and **1-P** (in the pathways leading to **2-E**) from the beginning of the processes up to the respective transition states and projected onto the key E···N bond-forming distance. Both reactions exhibit rather similar ASDs because the interaction between the deformed reactants becomes destabilizing in the early stages of the transformation, mainly due to increasing overlap between close shells of both approaching reactants, which causes steric (Pauli) repulsion. Despite that, the Δ*E*_int_ curve inverts at a certain point close to the transition state and becomes more and more stabilizing. This situation strongly resembles that found not only in related [3 + 2]-cycloaddition reactions^44^ but also in different pericyclic reactions including Diels–Alder, Alder-ene, and group transfer reactions.^20^ Moreover, the data in Figure 5 suggest that the interaction between the deformed reactants, although slightly more stabilizing for the process involving the heavier species **1-P**, does not constitute the ultimate factor governing the lower barrier computed for this reaction. At variance, it is the strain energy that is the dominant factor governing the enhanced reactivity of **1-P** as compared to **1-N**. As shown in Figure 5, the reaction involving the cyanide derivative exhibits a much more destabilizing strain than the analogous process involving its cyaphide counterpart, especially in the transition state region. This significantly increased strain is therefore responsible for the much higher barrier computed for the **1-N** + *t*BuN_3_ 1,3-dipolar cycloaddition reaction.

**Figure 5:**
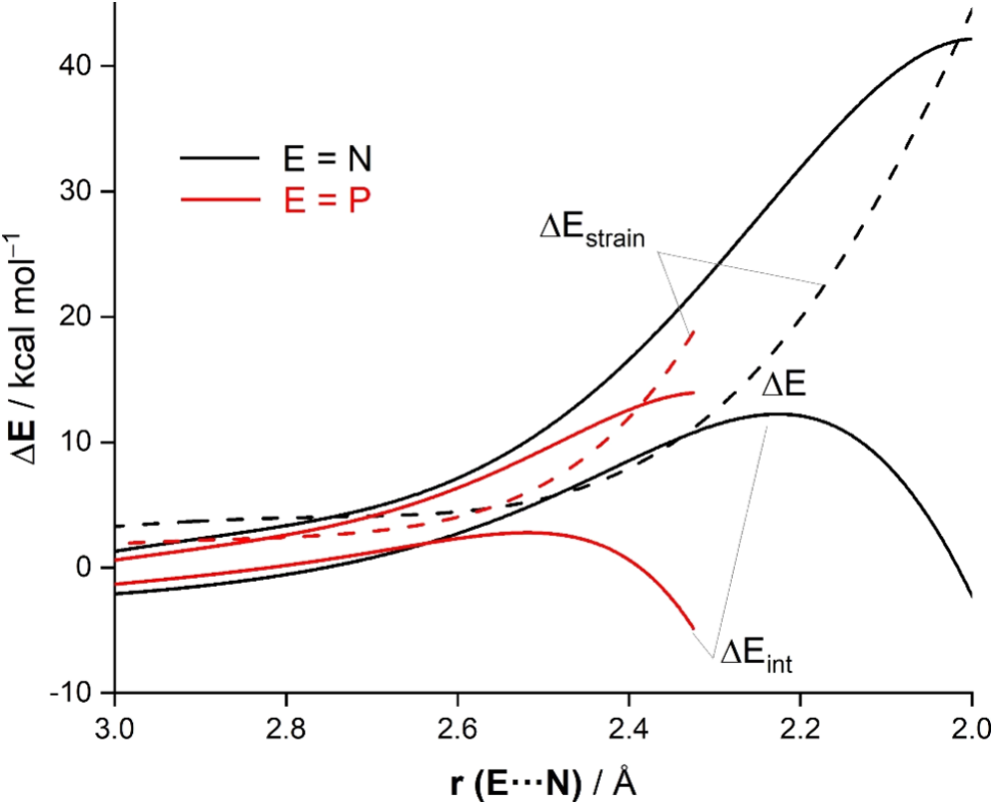
Activation strain analyses of the 1,3-dipolar cycloadditions between *t*BuN_3_ and **1-N** (black) and **1-P** (red) projected onto the E···N bond-forming distance. All data were computed at the PCM­(toluene)-M06-2X/def2-TZVPP//PCM­(toluene)-M06-2X/def2-SVP level.

To further confirm that the deformation energy is the dominant factor responsible for the computed reactivity trend (barrier height decreasing in the order **1-N** ≫ **1-P** > **1-As** > **1-Sb** ≥ **1-Bi**), we computed the corresponding activation strain values (i.e., at the respective transition states). Table 2 gathers the calculated values for the considered **1-E** + *t*BuN_3_ reactions leading to both regioisomers **2-E** and **2-iso-E**. From the data in Table 2, it is evident that the contribution of the interaction energy to the reactivity trend is almost negligible, as the Δ*E*_int_^‡^ values are rather similar for all reactions (spanning, for instance, from −5.5 kcal/mol for the process involving **1-N** to −8.2 kcal/mol for that involving **1-Bi**, for the reactions leading to the **2-E** regioisomer). At variance, the variation in the activation strain, Δ*E*_strain_^‡^, is much more pronounced: ranging from 45.0 kcal/mol for **1-N** to 10.7 kcal/mol for **1-Bi** (ΔΔ*E*_strain_^‡^ = 34.3 kcal/mol) in the preferred pathway.

**Table 2:** Computed Barriers and Activation Strain Values (in kcal/mol) for the 1,3-Cycloaddition Reactions Involving **1-E** + *t*BuN_3_

TS	Δ*G* ^‡^	Δ*E* ^‡^	Δ*E*_int_^‡^	Δ*E*_strain_^‡^	Δ*E*_strain_^‡^ (**1-E**)	Δ*E*_strain_^‡^ (azide)
CN	55.4	39.4	–5.5	45.0	8.9	36.1
CP	26.3	12.8	–5.9	18.7	2.5	16.2
CAs	23.4	9.6	–6.7	16.3	2.3	14.0
CSb	17.0	3.6	–7.3	10.9	1.1	9.8
CBi	16.7	2.5	–8.2	10.7	2.0	9.7
CN-iso	47.2	32.3	–6.7	39.0	7.2	31.9
CP-iso	28.2	14.6	–8.0	22.6	5.3	17.3
CAs-iso	25.1	11.3	–8.6	19.9	5.1	14.8
CSb-iso	21.7	6.9	–8.5	15.4	3.6	11.8
CBi-iso	20.4	6.2	–9.0	15.2	4.2	10.9

Data in Table 2 therefore confirm that the barrier heights and strain energies are strongly correlated. Indeed, a very good linear relationship was observed when plotting the Δ*G* ^‡^ values versus the total activation strain energies, Δ*E*_strain_^‡^ (correlation coefficient *R* ^2^ = 0.99, see Figure 6). Interestingly, the decomposition of these total strain values into the corresponding individual strain energies of both reactants indicates that the deformation of the azide reactant is much more significant (ranging from 36.1 to 9.7 kcal/mol, see Table 2) than that associated with the [Au^I^]-C ≡ E complex (i.e., the former contributing ca. 80–91% to the total Δ*E*_strain_^‡^). As a consequence, a strong linear correlation was also observed when plotting the barrier heights against the deformation energy of the azide reagent (correlation coefficient *R* ^2^ = 0.99, see Figure 6). At variance, the correlation found involving the strain of the **1-E** complexes not only is much poorer (*R* ^2^ = 0.82) but also exhibits a much lower slope, therefore indicating a much lower sensitivity of the barriers with respect to deformation required by the initial **1-E** complexes.

**Figure 6:**
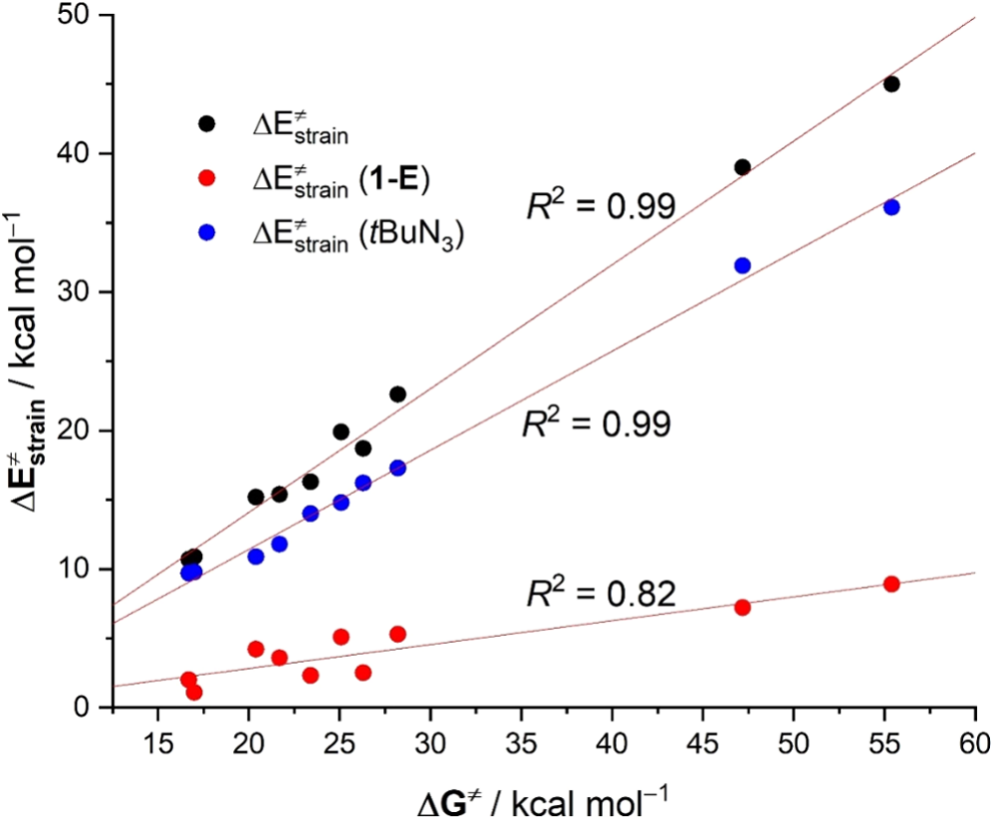
Plot of the computed free activation barriers for the **1-E** + *t*BuN_3_ 1,3-dipolar cycloadditions (Δ*G* ^‡^) versus the computed total strain energies (Δ*E*_strain_^‡^) and its contributors.

Thus, our calculations clearly suggest that the strain of the *t*BuN_3_ reactant, which progressively decreases down Group 15, constitutes the main factor governing the reactivity of the considered 1,3-dipolar cycloadditions. This indicates that the reactions involving the heavier complexes proceed earlier and earlier from **1-N** to **1-Bi**, and as a result, the energy required mainly to bend the *t*BuN_3_ reactant from its initial linear equilibrium geometry becomes lower and lower. This is not only confirmed by the different N–N–N angle in the corresponding transition states, which becomes larger and larger from **1-N** to **1-Bi** (132.8°, **TS-N**, < 144.6°, **TS-P**, < 146.8°, **TS-As**, < 150.7°, **TS-Sb**, < 152.0°, **TS-Bi**), but also by the Wiberg bond index of the key E···N bond-forming distance, which becomes lower and lower (WBI: 0.49, **TS-N** > 0.28, **TS-P** > 0.27, **TS-As** > 0.22, **TS-Sb** > 0.20, TS-Bi). Not surprisingly, both parameters also nicely correlate with the barrier heights: correlation coefficients of 0.98 and 0.99, for the N–N–N angle and WBIs, respectively (see Figures S2 and S3 in the Supporting Information), thus providing additional support for the key role of the strain (originating from the earlier/later nature of the transition states) in the transformation.

## Conclusions

The following conclusions can be drawn from this computational study: (i) the 1,3-dipolar cycloaddition reaction between the considered gold­(I) complexes **1-E** and *t*BuN_3_ is in all cases concerted and proceeds via a five-membered transition state which features in-plane aromaticity. (ii) While the process involving the cyanide complex can be considered unfeasible (barrier ca. 50 kcal/mol), the heavier systems lead to much lower activation barriers (ranging from ca. 17.0 to 26 kcal/mol). (iii) This indicates that the latter systems, in particular the heaviest Sb and Bi systems, constitute possible experimental targets for the preparation of heavy triazaphosphole analogues. (iv) With the exception of the cyanide complex, the process involving the heavier systems is regioselective toward the formation of the cyloadduct **2-E**, where the substituted nitrogen atom of the azide is attached to the E element. (v) This regioselectivity can be rationalized by the polarization induced by the E atom in the C ≡ E moiety, i.e., whereas the carbon atom shows a charge accumulation for E = P to Bi, the charge is mainly concentrated at the nitrogen atom in the cyanide complex as a consequence of its higher electronegativity. (vi) The trend in reactivity (N ≪ *P* < As < Sb < Bi) is mainly caused, according to the ASM approach, by the strain energy required by the reactants to reach the corresponding transition state geometries. In particular, the strain associated with the deformation of the azide dominates, which originates from the earlier and earlier nature of the transformation as going down in the Group 15. (vii) We hope that the present computational study may inspire future experimental research on the chemistry of these heavy main-group element-containing systems.

## Supplementary Material



## Notes

Cartesian coordinates and energies of all species discussed in the text (Figures S1–S3) (PDF)
