# Supplementary material for: Influence of the Nature of Group 15 Element on [AuI]–C≡E/Azide 1,3-Dipolar Cycloaddition Reaction
Source: Inorg Chem. 2025 Mar 12;64(11):5628–36. doi: 10.1021/acs.inorgchem.5c00110 (PMC12123619; doi:10.1021/acs.inorgchem.5c00110)
Supplement: Supplementary file 1 [file ic5c00110_si_001.pdf]

Supporting Information

Influence of the Nature of Group 15 Element on the [Au^{I}]−C≡E/azide 1,3-Dipolar Cycloaddition Reaction

*Daniel González-Pinardo and Israel Fernández**

Departamento de Química Orgánica and Centro de Innovación en Química Avanzada (ORFEO-CINQA), Facultad de Ciencias Químicas, Universidad Complutense de Madrid, 28040-Madrid (Spain)

e-mail: israel@quim.ucm.es

Contents

Figure S1……………………………………………………………………………………S2
Figure S2……………………………………………………………………………………S3
Figure S3……………………………………………………………………………………S3
Cartesian coordinates and energies…………………………………………………………S4

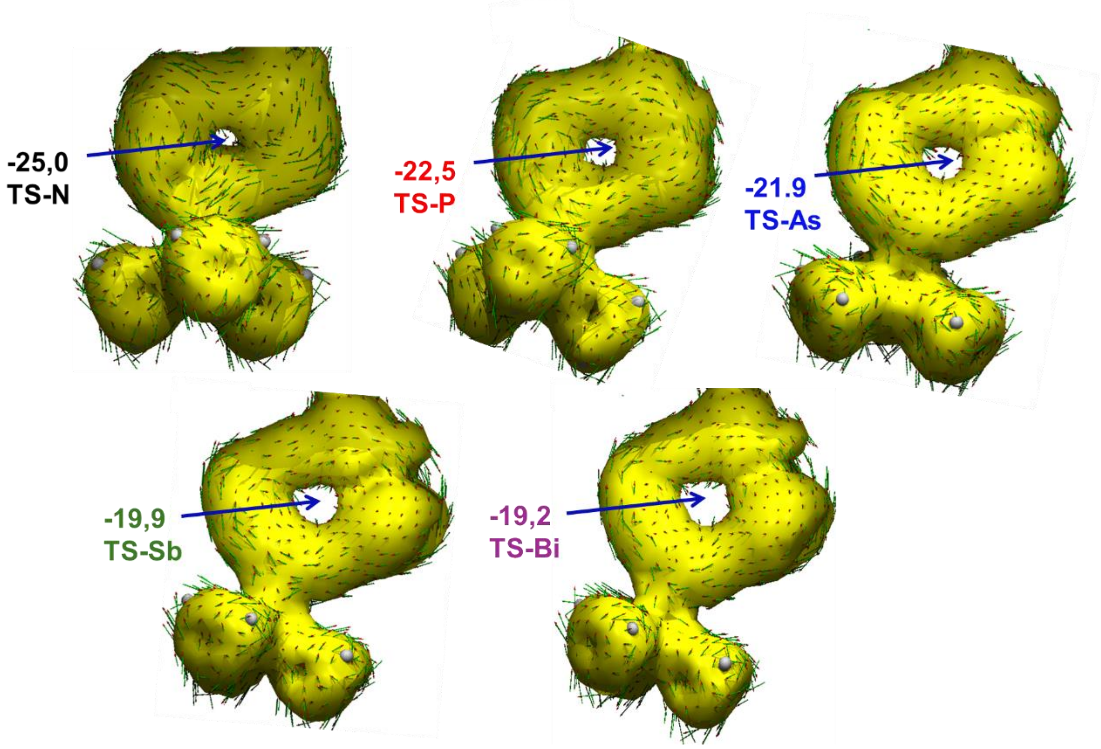

Figure S1. AICD plot (isosurface value of 0.015 a.u.) of **TS-N**. The values indicate the computed NICS(3,+1) values, in ppm, for **TS-N** (black), **TS-P** (red), **TS-As** (blue), **TS-Sb** (green) and **TS-Bi** (magenta).

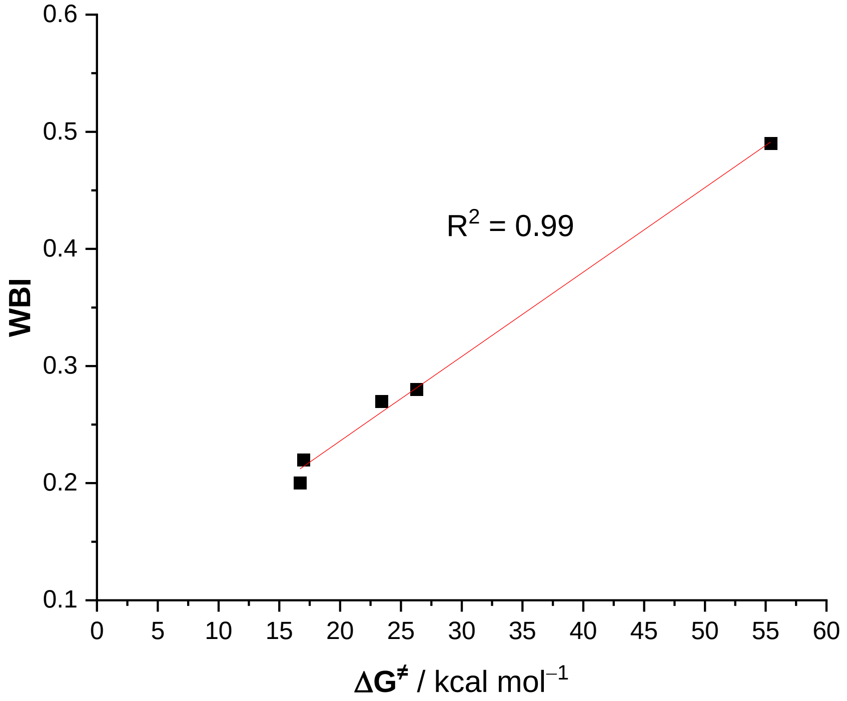

Figure S2. Plot of the computed free activation barriers for the **1-E** + *t*BuN_3 1,3-dipolar cycloadditions (ΔG≠) versus the respective Wiberg Bond Indexes.

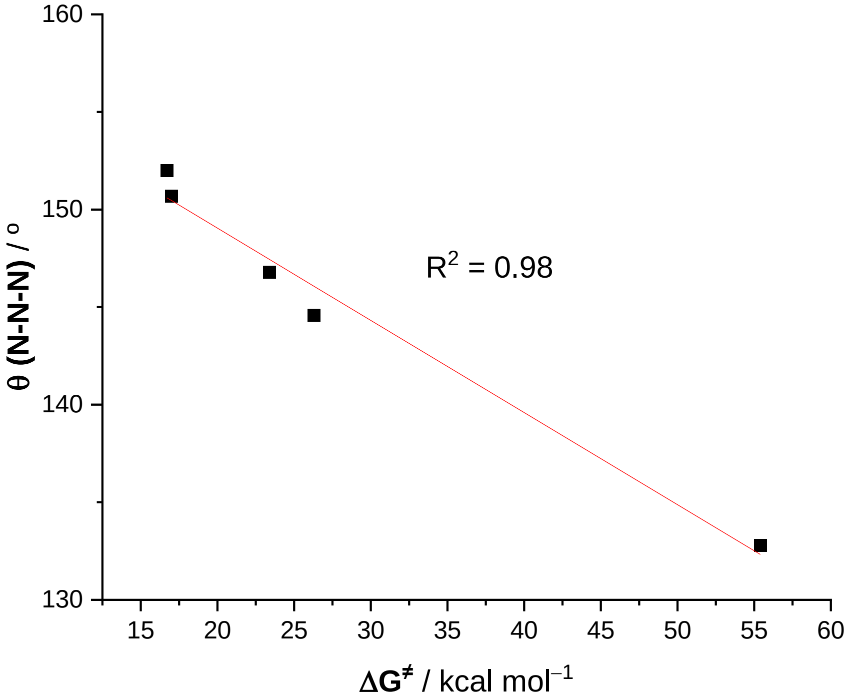

Figure S3. Plot of the computed free activation barriers for the **1-E** + *t*BuN_3 1,3-dipolar cycloadditions (ΔG≠) versus the respective azide angles (N-N-N).

Cartesian coordinates (in Å) and energies (in a.u.) of all the stationary points discussed in the text. All calculations have been performed at the PCM(toluene)-M06-2X/def2-SVP level.
SP denotes values computed at the PCM(toluene)-M06-2X/def2-TZVPP//PCM(toluene)-M06-2X/def2-SVP level.

1-N
***E* =** -1386.543771
***H* =** -1386.508238
***G* =** -1386.612379
G_{sp} = -1388.006444
N_{imag} = 0
***SP* =** -1388.531939

C	-4.263525000000	1.380326000000	-0.089149000000
C	-2.940308000000	1.340790000000	-0.542783000000
C	-2.431869000000	0.091981000000	-0.929912000000
C	-3.184783000000	-1.094934000000	-0.886251000000
C	-4.501275000000	-0.999691000000	-0.429169000000
C	-5.034556000000	0.224995000000	-0.032253000000
C	-2.121301000000	2.619665000000	-0.607745000000
C	-2.572129000000	-2.437371000000	-1.254671000000
C	-1.929054000000	-3.083374000000	-0.020015000000
C	-3.573993000000	-3.393139000000	-1.903514000000
C	-2.732849000000	3.606835000000	-1.607633000000
C	-1.968521000000	3.252721000000	0.778601000000
N	-1.069181000000	0.021516000000	-1.382974000000
C	-0.678495000000	0.019150000000	-2.709624000000
C	0.679309000000	-0.018661000000	-2.709459000000
N	1.069677000000	-0.021161000000	-1.382713000000
C	0.000156000000	0.000089000000	-0.557442000000
C	2.432280000000	-0.091789000000	-0.929424000000
C	2.940549000000	-1.340682000000	-0.542351000000
C	4.263748000000	-1.380410000000	-0.088670000000
C	5.034926000000	-0.225186000000	-0.031694000000
C	4.501812000000	0.999597000000	-0.428551000000
C	3.185343000000	1.095037000000	-0.885644000000
C	2.572826000000	2.437563000000	-1.253980000000
C	1.929281000000	3.083257000000	-0.019403000000
C	3.574912000000	3.393518000000	-1.902189000000
C	2.121433000000	-2.619475000000	-0.607501000000
C	2.732803000000	-3.606471000000	-1.607667000000
C	1.968733000000	-3.252823000000	0.778722000000
Au	-0.000365000000	-0.000120000000	1.507530000000
C	-0.000943000000	-0.000289000000	3.527413000000
N	-0.001225000000	-0.000345000000	4.687878000000
H	-4.693599000000	2.333401000000	0.224948000000
H	-5.119355000000	-1.896356000000	-0.375295000000
H	-6.063351000000	0.276264000000	0.326976000000
H	-1.112330000000	2.368084000000	-0.967303000000
H	-1.772421000000	-2.253060000000	-1.988344000000
H	-1.452228000000	-4.037611000000	-0.290134000000
H	-1.167114000000	-2.430123000000	0.431280000000
H	-2.693011000000	-3.283495000000	0.746517000000
H	-3.050845000000	-4.286344000000	-2.273064000000
H	-4.095061000000	-2.922468000000	-2.749471000000
H	-4.329859000000	-3.736111000000	-1.181876000000
H	-2.109954000000	4.510109000000	-1.681914000000
H	-2.816229000000	3.160312000000	-2.609099000000
H	-3.739554000000	3.917742000000	-1.289837000000
H	-1.332509000000	4.148085000000	0.720253000000

H	-1.510356000000	2.548718000000	1.489750000000
H	-2.944581000000	3.558782000000	1.184540000000
H	-1.398838000000	0.040843000000	-3.521608000000
H	1.399857000000	-0.040215000000	-3.521265000000
H	4.693695000000	-2.333560000000	0.225374000000
H	6.063712000000	-0.276611000000	0.327538000000
H	5.120011000000	1.896174000000	-0.374619000000
H	1.773402000000	2.253415000000	-1.988008000000
H	1.452566000000	4.037567000000	-0.289457000000
H	1.167167000000	2.429908000000	0.431449000000
H	2.692959000000	3.283178000000	0.747459000000
H	3.051869000000	4.286770000000	-2.271770000000
H	4.096369000000	2.923063000000	-2.748026000000
H	4.330448000000	3.736383000000	-1.180154000000
H	1.112444000000	-2.367750000000	-0.966900000000
H	2.109866000000	-4.509710000000	-1.682035000000
H	2.816047000000	-3.159752000000	-2.609058000000
H	3.739547000000	-3.917467000000	-1.290080000000
H	1.332569000000	-4.148070000000	0.720248000000
H	1.510768000000	-2.548917000000	1.490091000000
H	2.944793000000	-3.559152000000	1.184463000000

TS-N
***E* =** -1707.993760
***H* =** -1707.949863
***G* =** -1708.073527
G_{sp} = -1709.83405
N_{imag} = 1, -546 cm^{-1}
***SP* =** -1710.48771213

C	2.239481000000	-3.958548000000	-1.347589000000
C	2.340397000000	-2.564122000000	-1.288514000000
C	2.504624000000	-1.985652000000	-0.021209000000
C	2.571110000000	-2.735762000000	1.166367000000
C	2.470071000000	-4.124211000000	1.051541000000
C	2.304447000000	-4.729534000000	-0.192612000000
C	2.275214000000	-1.742559000000	-2.565565000000
C	2.674692000000	-2.061986000000	2.526015000000
C	1.273637000000	-1.769517000000	3.080254000000
C	3.495560000000	-2.870080000000	3.531862000000
C	3.455226000000	-2.071399000000	-3.486130000000
C	0.936203000000	-1.938578000000	-3.283238000000
N	2.596988000000	-0.554193000000	0.068963000000
C	3.780483000000	0.155722000000	0.167507000000
C	3.437635000000	1.469367000000	0.202075000000
N	2.058216000000	1.515612000000	0.106172000000
C	1.530621000000	0.274117000000	0.027625000000
C	1.271913000000	2.718236000000	0.145502000000
C	0.658717000000	3.073200000000	1.356374000000
C	-0.109418000000	4.243111000000	1.365136000000
C	-0.250166000000	5.015026000000	0.217749000000
C	0.371716000000	4.634387000000	-0.970017000000
C	1.145203000000	3.473198000000	-1.034330000000
C	1.763303000000	3.005241000000	-2.343011000000
C	0.798499000000	2.062895000000	-3.076015000000
C	2.185462000000	4.158853000000	-3.253213000000
C	0.812222000000	2.256237000000	2.628982000000
C	1.585815000000	3.049371000000	3.688086000000
C	-0.544491000000	1.787434000000	3.163393000000
Au	-0.461312000000	-0.232527000000	-0.099107000000
C	-2.439372000000	-0.736354000000	-0.206448000000
N	-3.268533000000	-1.279664000000	-0.888195000000

H	2.106250000000	-4.444992000000	-2.315899000000
H	2.513025000000	-4.743809000000	1.947786000000
H	2.221543000000	-5.815258000000	-0.259441000000
H	2.351735000000	-0.678303000000	-2.297115000000
H	3.185240000000	-1.096808000000	2.386608000000
H	1.344221000000	-1.250589000000	4.048128000000
H	0.685080000000	-1.142845000000	2.393163000000
H	0.720167000000	-2.708760000000	3.231152000000
H	3.654400000000	-2.278024000000	4.444209000000
H	4.478127000000	-3.147892000000	3.124229000000
H	2.974356000000	-3.791454000000	3.830655000000
H	3.424465000000	-1.440437000000	-4.386409000000
H	4.416454000000	-1.904170000000	-2.978814000000
H	3.421430000000	-3.122400000000	-3.810971000000
H	0.884260000000	-1.294719000000	-4.173478000000
H	0.090377000000	-1.688822000000	-2.624866000000
H	0.813242000000	-2.981078000000	-3.614004000000
H	4.746074000000	-0.338842000000	0.208082000000
H	4.039423000000	2.369443000000	0.281826000000
H	-0.604391000000	4.550896000000	2.288223000000
H	-0.854133000000	5.923147000000	0.245381000000
H	0.245630000000	5.248786000000	-1.861870000000
H	2.669892000000	2.429763000000	-2.100812000000
H	1.256551000000	1.690538000000	-4.004789000000
H	0.521145000000	1.198131000000	-2.454493000000
H	-0.128162000000	2.596149000000	-3.337311000000
H	2.748658000000	3.768223000000	-4.112431000000
H	2.820910000000	4.883023000000	-2.723550000000
H	1.313445000000	4.695711000000	-3.654798000000
H	1.401388000000	1.357053000000	2.395158000000
H	1.732317000000	2.438690000000	4.591015000000
H	2.573222000000	3.357763000000	3.314574000000
H	1.035262000000	3.956515000000	3.980560000000
H	-0.401097000000	1.153966000000	4.051238000000
H	-1.103769000000	1.206400000000	2.414183000000
H	-1.169540000000	2.642752000000	3.462411000000
N	-4.860965000000	-1.239011000000	0.311301000000
N	-4.379982000000	-0.493063000000	1.196392000000
N	-3.287971000000	-0.091548000000	1.400706000000
C	-6.143959000000	-0.891163000000	-0.323460000000
C	-6.357284000000	-1.939783000000	-1.412310000000
H	-5.524093000000	-1.913458000000	-2.125615000000
H	-6.401055000000	-2.943797000000	-0.967640000000
H	-7.300551000000	-1.742203000000	-1.941039000000
C	-7.257867000000	-0.974663000000	0.723384000000
H	-7.274990000000	-1.972469000000	1.183523000000
H	-7.101943000000	-0.227281000000	1.514333000000
H	-8.232676000000	-0.787138000000	0.249137000000
C	-6.065977000000	0.511788000000	-0.928551000000
H	-5.880317000000	1.261675000000	-0.144631000000
H	-5.244950000000	0.558734000000	-1.657496000000
H	-7.009515000000	0.765239000000	-1.433871000000

2-N

***E* =** -1708.080659
***H* =** -1708.037784
***G* =** -1708.160427
G_{sp} = -1709.913788
N_{imag} = 0
***SP* =** -1710.573564

C	0.253792000000	-4.306921000000	-1.365496000000
C	0.875463000000	-3.053650000000	-1.404051000000
C	1.531582000000	-2.625666000000	-0.240261000000
C	1.582730000000	-3.389734000000	0.939494000000
C	0.951300000000	-4.635549000000	0.923503000000
C	0.292814000000	-5.089753000000	-0.217552000000
C	0.823796000000	-2.220533000000	-2.674199000000
C	2.228229000000	-2.848201000000	2.205659000000
C	1.198966000000	-2.052617000000	3.020424000000
C	2.872469000000	-3.937486000000	3.063604000000
C	1.550104000000	-2.929616000000	-3.822090000000
C	-0.620213000000	-1.878172000000	-3.054957000000
N	2.173364000000	-1.340032000000	-0.249834000000
C	3.530409000000	-1.137139000000	-0.432143000000
C	3.723822000000	0.206581000000	-0.400167000000
N	2.475695000000	0.776014000000	-0.217872000000
C	1.513103000000	-0.167944000000	-0.120060000000
C	2.226373000000	2.187346000000	-0.108051000000
C	2.031337000000	2.736381000000	1.168196000000
C	1.790592000000	4.113177000000	1.242929000000
C	1.747876000000	4.895832000000	0.095197000000
C	1.937455000000	4.318988000000	-1.159229000000
C	2.175826000000	2.949113000000	-1.289624000000
C	2.290827000000	2.292936000000	-2.657008000000
C	0.910492000000	1.808014000000	-3.122187000000
C	2.927124000000	3.201092000000	-3.709310000000
C	2.083117000000	1.906291000000	2.440310000000
C	3.293700000000	2.300950000000	3.293498000000
C	0.779240000000	2.017856000000	3.237150000000
Au	-0.517432000000	0.134985000000	0.202066000000
C	-2.500786000000	0.449806000000	0.556038000000
N	-3.512900000000	0.127932000000	-0.257823000000
H	-0.269345000000	-4.671768000000	-2.251664000000
H	0.965757000000	-5.258887000000	1.818045000000
H	-0.198227000000	-6.063946000000	-0.207524000000
H	1.348127000000	-1.271806000000	-2.487528000000
H	3.025952000000	-2.151196000000	1.906971000000
H	1.670449000000	-1.614142000000	3.912946000000
H	0.750706000000	-1.240371000000	2.428413000000
H	0.382502000000	-2.713037000000	3.349982000000
H	3.436849000000	-3.477904000000	3.887247000000
H	3.562368000000	-4.561473000000	2.477452000000
H	2.115709000000	-4.594447000000	3.517098000000
H	1.547604000000	-2.298458000000	-4.722866000000
H	2.594315000000	-3.151037000000	-3.557652000000
H	1.055727000000	-3.879654000000	-4.075942000000
H	-0.634466000000	-1.228000000000	-3.942170000000
H	-1.138115000000	-1.356583000000	-2.235311000000
H	-1.191683000000	-2.787766000000	-3.295389000000
H	4.224340000000	-1.962046000000	-0.561452000000
H	4.623249000000	0.807359000000	-0.494257000000
H	1.632533000000	4.575827000000	2.219006000000
H	1.558400000000	5.967235000000	0.175421000000
H	1.890055000000	4.945688000000	-2.050195000000
H	2.936600000000	1.407588000000	-2.554234000000
H	0.991407000000	1.284728000000	-4.087112000000
H	0.450838000000	1.124773000000	-2.391939000000
H	0.230840000000	2.664488000000	-3.248928000000
H	3.108997000000	2.631879000000	-4.631855000000
H	3.885486000000	3.614361000000	-3.363468000000
H	2.265665000000	4.039925000000	-3.971538000000

H	2.206820000000	0.850151000000	2.159523000000
H	3.349146000000	1.670330000000	4.192995000000
H	4.232593000000	2.186924000000	2.732096000000
H	3.219175000000	3.349360000000	3.620583000000
H	0.815299000000	1.353694000000	4.113301000000
H	-0.095615000000	1.738446000000	2.629851000000
H	0.624944000000	3.044192000000	3.603643000000
N	-4.580915000000	0.520329000000	0.407411000000
N	-4.302360000000	1.059588000000	1.565247000000
N	-3.009832000000	1.028993000000	1.686897000000
C	-5.947528000000	0.364554000000	-0.131550000000
C	-6.173490000000	-1.123324000000	-0.403542000000
H	-5.427412000000	-1.498564000000	-1.115918000000
H	-6.091403000000	-1.699693000000	0.528961000000
H	-7.178315000000	-1.273045000000	-0.822916000000
C	-6.942591000000	0.885133000000	0.899403000000
H	-6.862572000000	0.326802000000	1.840812000000
H	-6.767725000000	1.946358000000	1.116490000000
H	-7.957485000000	0.763910000000	0.496038000000
C	-6.030349000000	1.173249000000	-1.427088000000
H	-5.847866000000	2.238369000000	-1.225309000000
H	-5.280790000000	0.817452000000	-2.145973000000
H	-7.031150000000	1.064403000000	-1.868146000000

TS-iso-N
***E* =** -1708.007108
***H* =** -1707.963386
***G* =** -1708.085432
G_{sp} = -1709.847222
N_{imag} = 1, -463 cm^{-1}
***SP* =** -1710.50145108

C	-4.687053000000	-1.691877000000	0.501585000000
C	-3.735178000000	-0.754226000000	0.912784000000
C	-3.229667000000	0.118671000000	-0.063532000000
C	-3.624584000000	0.080270000000	-1.409763000000
C	-4.579535000000	-0.875568000000	-1.769318000000
C	-5.106904000000	-1.750742000000	-0.824213000000
C	-3.246786000000	-0.734962000000	2.351856000000
C	-3.014909000000	0.990960000000	-2.462579000000
C	-2.109862000000	0.184692000000	-3.401143000000
C	-4.086126000000	1.756200000000	-3.244192000000
C	-4.406396000000	-0.599684000000	3.342546000000
C	-2.400701000000	-1.978319000000	2.649607000000
N	-2.236387000000	1.079734000000	0.329799000000
C	-2.510318000000	2.331934000000	0.847578000000
C	-1.308366000000	2.907921000000	1.109705000000
N	-0.343367000000	1.990930000000	0.732328000000
C	-0.907488000000	0.856228000000	0.258061000000
C	1.073632000000	2.204994000000	0.853371000000
C	1.706046000000	3.013458000000	-0.109962000000
C	3.078513000000	3.227802000000	0.033320000000
C	3.788100000000	2.646197000000	1.082258000000
C	3.137751000000	1.838773000000	2.007798000000
C	1.760614000000	1.600608000000	1.917344000000
C	1.074467000000	0.736621000000	2.962534000000
C	1.739274000000	-0.638147000000	3.080969000000
C	1.036854000000	1.458619000000	4.314080000000
C	0.940117000000	3.562112000000	-1.304113000000
C	1.517029000000	4.870876000000	-1.842779000000
C	0.875127000000	2.503061000000	-2.413476000000

Au	0.034507000000	-0.905504000000	-0.236862000000
C	0.948730000000	-2.700073000000	-0.540932000000
N	0.892974000000	-3.895968000000	-0.507056000000
H	-5.096673000000	-2.395253000000	1.228977000000
H	-4.906535000000	-0.942842000000	-2.808678000000
H	-5.846122000000	-2.493955000000	-1.126278000000
H	-2.599261000000	0.144911000000	2.482492000000
H	-2.384109000000	1.734453000000	-1.951607000000
H	-1.629713000000	0.847169000000	-4.136342000000
H	-1.323167000000	-0.341489000000	-2.838893000000
H	-2.693091000000	-0.569931000000	-3.950709000000
H	-3.613562000000	2.451070000000	-3.953420000000
H	-4.735116000000	2.335920000000	-2.572260000000
H	-4.722444000000	1.072233000000	-3.825311000000
H	-4.020887000000	-0.524717000000	4.369551000000
H	-5.010141000000	0.295155000000	3.133278000000
H	-5.071146000000	-1.475381000000	3.301690000000
H	-2.011894000000	-1.938694000000	3.678094000000
H	-1.548346000000	-2.058252000000	1.957353000000
H	-3.003008000000	-2.893834000000	2.548380000000
H	-3.526384000000	2.689484000000	0.982591000000
H	-1.049220000000	3.878425000000	1.521883000000
H	3.605708000000	3.849973000000	-0.690211000000
H	4.861160000000	2.821459000000	1.173906000000
H	3.705921000000	1.385427000000	2.822339000000
H	0.035144000000	0.570003000000	2.644796000000
H	1.172375000000	-1.273831000000	3.776523000000
H	1.778888000000	-1.149456000000	2.106483000000
H	2.766119000000	-0.554988000000	3.468851000000
H	0.507440000000	0.847729000000	5.059763000000
H	0.524584000000	2.428511000000	4.234096000000
H	2.055498000000	1.642815000000	4.688424000000
H	-0.091496000000	3.767249000000	-0.980378000000
H	0.844659000000	5.287205000000	-2.605897000000
H	1.641158000000	5.618864000000	-1.046667000000
H	2.494111000000	4.713856000000	-2.323032000000
H	0.282317000000	2.873055000000	-3.263536000000
H	0.422886000000	1.564728000000	-2.057142000000
H	1.888961000000	2.270726000000	-2.774091000000
N	2.630991000000	-4.621932000000	-0.679451000000
N	3.273321000000	-3.639179000000	-0.786331000000
N	2.951297000000	-2.423192000000	-0.797212000000
C	3.982937000000	-1.392966000000	-0.990076000000
C	3.241652000000	-0.087412000000	-1.269304000000
H	2.643358000000	0.218856000000	-0.397231000000
H	3.962269000000	0.714889000000	-1.485841000000
H	2.567386000000	-0.206559000000	-2.130677000000
C	4.824311000000	-1.255197000000	0.282468000000
H	5.572675000000	-0.455661000000	0.169409000000
H	4.175870000000	-1.009868000000	1.136423000000
H	5.347902000000	-2.198159000000	0.499343000000
C	4.869768000000	-1.750670000000	-2.186146000000
H	4.257886000000	-1.863496000000	-3.092710000000
H	5.614625000000	-0.960003000000	-2.359927000000
H	5.399225000000	-2.696701000000	-2.004289000000

2-iso-N
E **=** -1708.087318
H **=** -1708.044832
G **=** -1708.163085
G_{sp} **=** -1709.92067

***N_{imag}* =** 0
***SP* =** -1710.582442

C	4.277127000000	1.818154000000	1.190418000000
C	3.319097000000	0.803820000000	1.302237000000
C	3.169548000000	-0.060389000000	0.207491000000
C	3.923671000000	0.054799000000	-0.974432000000
C	4.869291000000	1.080547000000	-1.032243000000
C	5.044257000000	1.953856000000	0.039769000000
C	2.506772000000	0.667967000000	2.579992000000
C	3.664645000000	-0.853951000000	-2.166333000000
C	2.505159000000	-0.300706000000	-3.006310000000
C	4.901923000000	-1.073739000000	-3.036498000000
C	3.407313000000	0.265147000000	3.752888000000
C	1.729946000000	1.951960000000	2.887286000000
N	2.195403000000	-1.114490000000	0.295998000000
C	2.486848000000	-2.432051000000	0.602914000000
C	1.299625000000	-3.091440000000	0.639084000000
N	0.325155000000	-2.148271000000	0.367619000000
C	0.865006000000	-0.927944000000	0.153618000000
C	-1.087866000000	-2.395158000000	0.299737000000
C	-1.665641000000	-2.600831000000	-0.962706000000
C	-3.054670000000	-2.760038000000	-1.011920000000
C	-3.820171000000	-2.724244000000	0.149531000000
C	-3.212772000000	-2.538876000000	1.389526000000
C	-1.829303000000	-2.369465000000	1.493433000000
C	-1.170629000000	-2.119258000000	2.841715000000
C	-1.304031000000	-0.647186000000	3.250844000000
C	-1.717063000000	-3.039840000000	3.935324000000
C	-0.840205000000	-2.667034000000	-2.238389000000
C	-1.008801000000	-4.027559000000	-2.923420000000
C	-1.181412000000	-1.519563000000	-3.193238000000
Au	-0.187049000000	0.821475000000	-0.202093000000
C	-1.224628000000	2.567331000000	-0.478192000000
N	-0.650343000000	3.761750000000	-0.618911000000
H	4.420495000000	2.512585000000	2.020387000000
H	5.474629000000	1.206882000000	-1.929946000000
H	5.784349000000	2.752425000000	-0.028004000000
H	1.768542000000	-0.135484000000	2.438862000000
H	3.356176000000	-1.837651000000	-1.779740000000
H	2.288322000000	-0.971189000000	-3.851582000000
H	1.588149000000	-0.185384000000	-2.409587000000
H	2.767049000000	0.689767000000	-3.408024000000
H	4.692965000000	-1.843359000000	-3.792800000000
H	5.765990000000	-1.400440000000	-2.440304000000
H	5.182782000000	-0.157181000000	-3.575893000000
H	2.808917000000	0.129665000000	4.665817000000
H	3.938068000000	-0.675141000000	3.543848000000
H	4.160324000000	1.041928000000	3.955318000000
H	1.099356000000	1.809209000000	3.777212000000
H	1.082355000000	2.239037000000	2.044729000000
H	2.412026000000	2.790888000000	3.091638000000
H	3.504972000000	-2.773640000000	0.762993000000
H	1.058648000000	-4.131763000000	0.834766000000
H	-3.542872000000	-2.912462000000	-1.976843000000
H	-4.902892000000	-2.846029000000	0.089575000000
H	-3.827092000000	-2.515931000000	2.291046000000
H	-0.096946000000	-2.336177000000	2.738046000000
H	-0.798727000000	-0.471283000000	4.212481000000
H	-0.860307000000	0.024922000000	2.499642000000
H	-2.364285000000	-0.372633000000	3.364927000000
H	-1.132651000000	-2.913171000000	4.857700000000

H	-1.666618000000	-4.096404000000	3.635902000000
H	-2.763316000000	-2.802138000000	4.177623000000
H	0.221291000000	-2.563039000000	-1.967269000000
H	-0.356817000000	-4.091997000000	-3.806488000000
H	-0.752549000000	-4.852017000000	-2.242628000000
H	-2.045463000000	-4.176224000000	-3.261320000000
H	-0.581790000000	-1.598337000000	-4.111856000000
H	-0.973496000000	-0.542683000000	-2.731357000000
H	-2.244605000000	-1.543390000000	-3.478849000000
N	-1.627152000000	4.670872000000	-0.742403000000
N	-2.774874000000	4.113572000000	-0.686237000000
N	-2.563766000000	2.805072000000	-0.520702000000
C	-3.696215000000	1.856087000000	-0.424446000000
C	-3.616924000000	0.872488000000	-1.593457000000
H	-2.702077000000	0.266727000000	-1.530917000000
H	-4.479467000000	0.190348000000	-1.561745000000
H	-3.622982000000	1.410426000000	-2.552609000000
C	-3.607525000000	1.104853000000	0.905978000000
H	-4.488789000000	0.456494000000	1.020844000000
H	-2.712446000000	0.466900000000	0.941899000000
H	-3.574131000000	1.811912000000	1.747894000000
C	-5.005081000000	2.641950000000	-0.490353000000
H	-5.089406000000	3.194216000000	-1.434436000000
H	-5.839867000000	1.931354000000	-0.414168000000
H	-5.073684000000	3.365290000000	0.331899000000

1-P
***E* =** -1673.038806
***H* =** -1673.002908
***G* =** -1673.108886
G_{sp} = -1674.549182
N_{imag} = 0
***SP* =** -1675.071162

C	-4.271137000000	1.384009000000	-0.192693000000
C	-2.946451000000	1.366374000000	-0.643352000000
C	-2.429946000000	0.134401000000	-1.071395000000
C	-3.177006000000	-1.056929000000	-1.069540000000
C	-4.495323000000	-0.984181000000	-0.613511000000
C	-5.036469000000	0.223592000000	-0.177439000000
C	-2.132836000000	2.650162000000	-0.661364000000
C	-2.556201000000	-2.383124000000	-1.480558000000
C	-1.919817000000	-3.070130000000	-0.264744000000
C	-3.549501000000	-3.318813000000	-2.170676000000
C	-2.748705000000	3.671298000000	-1.623727000000
C	-1.981538000000	3.231257000000	0.747700000000
N	-1.066784000000	0.086325000000	-1.524010000000
C	-0.675681000000	0.133514000000	-2.850083000000
C	0.681935000000	0.096235000000	-2.850070000000
N	1.070559000000	0.044648000000	-1.523407000000
C	0.000985000000	0.034828000000	-0.696469000000
C	2.432679000000	-0.047181000000	-1.074135000000
C	2.936862000000	-1.310795000000	-0.732190000000
C	4.261870000000	-1.372642000000	-0.286338000000
C	5.038930000000	-0.223804000000	-0.192367000000
C	4.509404000000	1.016680000000	-0.542517000000
C	3.191338000000	1.133828000000	-0.989913000000
C	2.581461000000	2.491087000000	-1.304439000000
C	1.938105000000	3.087107000000	-0.044987000000
C	3.585048000000	3.470882000000	-1.913415000000
C	2.111143000000	-2.582563000000	-0.837493000000
C	2.702055000000	-3.528837000000	-1.888029000000

C	1.976999000000	-3.272236000000	0.523458000000
Au	-0.001491000000	-0.050536000000	1.382502000000
C	-0.003760000000	-0.139512000000	3.377290000000
P	-0.005288000000	-0.214412000000	4.932877000000
H	-4.706651000000	2.323850000000	0.152414000000
H	-5.108742000000	-1.885436000000	-0.591785000000
H	-6.066676000000	0.257425000000	0.179919000000
H	-1.123183000000	2.415387000000	-1.030115000000
H	-1.751672000000	-2.169586000000	-2.200877000000
H	-1.435913000000	-4.011662000000	-0.565567000000
H	-1.165794000000	-2.429528000000	0.217024000000
H	-2.689253000000	-3.301829000000	0.487386000000
H	-3.020508000000	-4.197125000000	-2.566776000000
H	-4.065386000000	-2.820606000000	-3.003981000000
H	-4.310159000000	-3.688871000000	-1.467585000000
H	-2.129060000000	4.578947000000	-1.665233000000
H	-2.831422000000	3.261734000000	-2.640967000000
H	-3.756322000000	3.966876000000	-1.294203000000
H	-1.350160000000	4.131541000000	0.722115000000
H	-1.518663000000	2.502600000000	1.430374000000
H	-2.958969000000	3.516890000000	1.165373000000
H	-1.395600000000	0.184807000000	-3.661157000000
H	1.403184000000	0.105302000000	-3.661515000000
H	4.688532000000	-2.338261000000	-0.007850000000
H	6.069235000000	-0.292598000000	0.159683000000
H	5.131658000000	1.908197000000	-0.459090000000
H	1.782011000000	2.337367000000	-2.045435000000
H	1.461218000000	4.051349000000	-0.277074000000
H	1.177237000000	2.415837000000	0.380957000000
H	2.702591000000	3.256854000000	0.728387000000
H	3.063424000000	4.378859000000	-2.247528000000
H	4.106589000000	3.033904000000	-2.777043000000
H	4.340572000000	3.783903000000	-1.177831000000
H	1.097669000000	-2.311569000000	-1.168784000000
H	2.075918000000	-4.427455000000	-1.988228000000
H	2.768347000000	-3.041696000000	-2.871726000000
H	3.713859000000	-3.853698000000	-1.601541000000
H	1.331027000000	-4.158394000000	0.438646000000
H	1.538211000000	-2.593606000000	1.270520000000
H	2.956914000000	-3.605018000000	0.898142000000

TS-P
E **=** -1994.533151
H **=** -1994.488364
G **=** -1994.615291
G_{sp} **=** -1996.423221
N_{imag} **=** 1, -371 cm^{-1}
SP **=** -1997.072078

C	1.727335000000	-4.317133000000	-1.109948000000
C	2.168038000000	-3.003425000000	-0.920293000000
C	1.984529000000	-2.440775000000	0.351899000000
C	1.380163000000	-3.128430000000	1.417503000000
C	0.956288000000	-4.438099000000	1.175883000000
C	1.129070000000	-5.027256000000	-0.074052000000
C	2.789846000000	-2.232918000000	-2.073140000000
C	1.128778000000	-2.466064000000	2.762667000000
C	-0.331560000000	-2.007718000000	2.866264000000
C	1.505989000000	-3.372114000000	3.937002000000
C	4.045117000000	-2.935198000000	-2.599663000000
C	1.764700000000	-2.008385000000	-3.189664000000
N	2.407610000000	-1.085277000000	0.566531000000

C	3.626680000000	-0.711686000000	1.103161000000
C	3.626737000000	0.645914000000	1.140877000000
N	2.413385000000	1.051506000000	0.613140000000
C	1.650310000000	-0.008508000000	0.260987000000
C	1.983964000000	2.418384000000	0.504869000000
C	1.115313000000	2.925474000000	1.483049000000
C	0.710880000000	4.258874000000	1.350641000000
C	1.157502000000	5.040571000000	0.291646000000
C	2.018797000000	4.506778000000	-0.665019000000
C	2.448994000000	3.180610000000	-0.581991000000
C	3.322904000000	2.562686000000	-1.662667000000
C	2.445709000000	1.896313000000	-2.731547000000
C	4.285587000000	3.563321000000	-2.302241000000
C	0.629372000000	2.099506000000	2.663278000000
C	1.211669000000	2.640842000000	3.973697000000
C	-0.900056000000	2.036800000000	2.714441000000
Au	-0.278376000000	0.003506000000	-0.485656000000
C	-2.168152000000	0.010296000000	-1.168677000000
P	-3.426440000000	0.078211000000	-2.129946000000
H	1.847285000000	-4.786640000000	-2.088328000000
H	0.472770000000	-5.002560000000	1.974759000000
H	0.786097000000	-6.048949000000	-0.243652000000
H	3.094908000000	-1.243037000000	-1.701628000000
H	1.763814000000	-1.569424000000	2.823733000000
H	-0.506241000000	-1.501645000000	3.827842000000
H	-0.601764000000	-1.315169000000	2.054631000000
H	-1.011105000000	-2.871769000000	2.805627000000
H	1.405472000000	-2.821708000000	4.883428000000
H	2.542075000000	-3.730585000000	3.853579000000
H	0.844951000000	-4.249286000000	3.997778000000
H	4.507936000000	-2.338868000000	-3.399414000000
H	4.787584000000	-3.078957000000	-1.801361000000
H	3.801405000000	-3.923298000000	-3.018455000000
H	2.204770000000	-1.405134000000	-3.997380000000
H	0.873732000000	-1.485360000000	-2.810058000000
H	1.438594000000	-2.967063000000	-3.621326000000
H	4.369888000000	-1.441849000000	1.408134000000
H	4.370517000000	1.357247000000	1.486673000000
H	0.034556000000	4.688655000000	2.092088000000
H	0.829785000000	6.077697000000	0.206338000000
H	2.354774000000	5.132237000000	-1.492430000000
H	3.931789000000	1.774523000000	-1.193728000000
H	3.072558000000	1.413305000000	-3.496290000000
H	1.778754000000	1.137238000000	-2.296296000000
H	1.814304000000	2.649346000000	-3.227192000000
H	4.982211000000	3.035661000000	-2.968877000000
H	4.872972000000	4.103580000000	-1.545917000000
H	3.749630000000	4.303410000000	-2.914666000000
H	0.993424000000	1.069045000000	2.538321000000
H	0.892188000000	2.016622000000	4.821066000000
H	2.311133000000	2.652877000000	3.947688000000
H	0.865965000000	3.668814000000	4.162150000000
H	-1.224381000000	1.391295000000	3.543989000000
H	-1.315472000000	1.625746000000	1.782157000000
H	-1.334002000000	3.035170000000	2.877503000000
N	-4.980481000000	-0.359710000000	-0.457183000000
N	-4.253642000000	-0.348600000000	0.556615000000
N	-3.164592000000	-0.325175000000	0.931634000000
C	-6.371953000000	0.125367000000	-0.368160000000
C	-6.953182000000	-0.021952000000	-1.772019000000
H	-6.403508000000	0.603685000000	-2.488956000000

H	-6.887985000000	-1.067559000000	-2.104432000000
H	-8.007924000000	0.286585000000	-1.771866000000
C	-7.152198000000	-0.743469000000	0.620588000000
H	-7.117221000000	-1.797615000000	0.311870000000
H	-6.722922000000	-0.660035000000	1.629784000000
H	-8.203081000000	-0.421669000000	0.661667000000
C	-6.383088000000	1.592379000000	0.067473000000
H	-5.949779000000	1.700300000000	1.073365000000
H	-5.790658000000	2.199480000000	-0.632286000000
H	-7.411953000000	1.980391000000	0.090133000000

2-P
***E* =** -1994.637401
***H* =** -1994.593780
***G* =** -1994.718818
G_{sp} = -1996.523711
N_{imag} = 0
***SP* =** -1997.177664

C	1.481829000000	4.310467000000	1.244330000000
C	1.887763000000	2.971861000000	1.229596000000
C	2.136240000000	2.388735000000	-0.022159000000
C	1.993637000000	3.082619000000	-1.235872000000
C	1.588051000000	4.418136000000	-1.166493000000
C	1.334695000000	5.026239000000	0.060698000000
C	2.019070000000	2.199199000000	2.531566000000
C	2.198489000000	2.398386000000	-2.578146000000
C	0.848472000000	1.955680000000	-3.157090000000
C	2.962138000000	3.273623000000	-3.573998000000
C	3.013294000000	2.870663000000	3.483625000000
C	0.648571000000	2.018069000000	3.192864000000
N	2.537910000000	1.010726000000	-0.067922000000
C	3.847687000000	0.574624000000	-0.168320000000
C	3.796932000000	-0.782211000000	-0.191948000000
N	2.460220000000	-1.124846000000	-0.089584000000
C	1.673699000000	-0.026461000000	-0.018313000000
C	1.955566000000	-2.471005000000	-0.103146000000
C	1.381833000000	-2.961259000000	-1.286170000000
C	0.905861000000	-4.277583000000	-1.268287000000
C	1.000303000000	-5.058074000000	-0.122468000000
C	1.566070000000	-4.538555000000	1.040432000000
C	2.050995000000	-3.229514000000	1.078064000000
C	2.581348000000	-2.616846000000	2.365613000000
C	1.458311000000	-1.853689000000	3.082373000000
C	3.220403000000	-3.640181000000	3.303779000000
C	1.287096000000	-2.139112000000	-2.561352000000
C	2.268951000000	-2.670988000000	-3.611905000000
C	-0.143793000000	-2.094807000000	-3.106783000000
Au	-0.407293000000	-0.001497000000	0.009877000000
C	-2.447336000000	-0.037696000000	-0.052583000000
P	-3.551968000000	0.707852000000	1.037864000000
H	1.272495000000	4.796540000000	2.199331000000
H	1.458337000000	4.989365000000	-2.086896000000
H	1.013822000000	6.068482000000	0.092981000000
H	2.408852000000	1.196768000000	2.299314000000
H	2.799849000000	1.492406000000	-2.409540000000
H	0.995024000000	1.420274000000	-4.107284000000
H	0.309256000000	1.292819000000	-2.462898000000
H	0.208603000000	2.830423000000	-3.350075000000
H	3.181417000000	2.699727000000	-4.485651000000
H	3.913057000000	3.629862000000	-3.152442000000

H	2.371597000000	4.151115000000	-3.876068000000
H	3.135187000000	2.265900000000	4.394013000000
H	3.999779000000	2.989476000000	3.012750000000
H	2.661053000000	3.866978000000	3.790471000000
H	0.741574000000	1.418020000000	4.110095000000
H	-0.053000000000	1.509261000000	2.513971000000
H	0.213440000000	2.991854000000	3.465596000000
H	4.683213000000	1.265903000000	-0.218825000000
H	4.579555000000	-1.531003000000	-0.267641000000
H	0.452971000000	-4.694003000000	-2.169798000000
H	0.623078000000	-6.081754000000	-0.130455000000
H	1.621762000000	-5.161147000000	1.933602000000
H	3.360146000000	-1.886726000000	2.097251000000
H	1.843375000000	-1.365640000000	3.990591000000
H	1.010541000000	-1.084388000000	2.435472000000
H	0.656770000000	-2.548507000000	3.375967000000
H	3.704064000000	-3.123323000000	4.144614000000
H	3.979069000000	-4.246586000000	2.788428000000
H	2.468474000000	-4.320164000000	3.730702000000
H	1.578970000000	-1.104801000000	-2.328460000000
H	2.227634000000	-2.054157000000	-4.521787000000
H	3.302264000000	-2.662887000000	-3.234781000000
H	2.018807000000	-3.705739000000	-3.892507000000
H	-0.189046000000	-1.426619000000	-3.979574000000
H	-0.863142000000	-1.728217000000	-2.357488000000
H	-0.474670000000	-3.090987000000	-3.438094000000
N	-4.815537000000	0.044350000000	0.076598000000
N	-4.397302000000	-0.661335000000	-0.953447000000
N	-3.112440000000	-0.717245000000	-1.039649000000
C	-6.285428000000	0.151663000000	0.250152000000
C	-6.580355000000	1.008865000000	1.478188000000
H	-6.158417000000	0.559736000000	2.388894000000
H	-6.169638000000	2.022574000000	1.364679000000
H	-7.669083000000	1.088845000000	1.604673000000
C	-6.872468000000	0.807325000000	-1.001512000000
H	-6.441976000000	1.809277000000	-1.143438000000
H	-6.649986000000	0.200815000000	-1.888519000000
H	-7.962546000000	0.903988000000	-0.896123000000
C	-6.851493000000	-1.256796000000	0.443942000000
H	-6.627023000000	-1.880744000000	-0.430567000000
H	-6.407008000000	-1.725779000000	1.333833000000
H	-7.941401000000	-1.206569000000	0.579386000000

TS-iso-P
***E* =** -1994.530290
***H* =** -1994.485831
***G* =** -1994.610239
G_{sp} = -1996.420183
N_{imag} = 1, -368 cm^{-1}
***SP* =** -1997.070222

C	4.470575000000	-1.119908000000	-1.826936000000
C	3.560689000000	-0.100206000000	-1.532149000000
C	3.237969000000	0.103325000000	-0.181337000000
C	3.773495000000	-0.668675000000	0.861718000000
C	4.678935000000	-1.675499000000	0.514186000000
C	5.025643000000	-1.898159000000	-0.815231000000
C	2.920202000000	0.704785000000	-2.650726000000
C	3.362529000000	-0.473424000000	2.311598000000
C	2.507187000000	-1.652342000000	2.790383000000
C	4.575444000000	-0.259772000000	3.221395000000

C	3.967779000000	1.338190000000	-3.570050000000
C	1.941662000000	-0.171913000000	-3.440482000000
N	2.293489000000	1.135041000000	0.146521000000
C	2.631043000000	2.430228000000	0.494028000000
C	1.461690000000	3.077736000000	0.735774000000
N	0.452670000000	2.156585000000	0.516468000000
C	0.955403000000	0.951128000000	0.161184000000
C	-0.950774000000	2.418935000000	0.678039000000
C	-1.591053000000	1.952688000000	1.836793000000
C	-2.964608000000	2.198911000000	1.951009000000
C	-3.651748000000	2.896689000000	0.964263000000
C	-2.983552000000	3.360692000000	-0.167274000000
C	-1.618686000000	3.121822000000	-0.341720000000
C	-0.897655000000	3.533722000000	-1.616100000000
C	-0.882183000000	2.366294000000	-2.612444000000
C	-1.485806000000	4.786526000000	-2.264452000000
C	-0.843841000000	1.270525000000	2.972189000000
C	-0.756892000000	2.213643000000	4.178528000000
C	-1.467761000000	-0.071912000000	3.359759000000
Au	-0.060075000000	-0.838187000000	-0.082368000000
C	-0.949098000000	-2.642663000000	-0.158113000000
P	-0.916887000000	-4.213466000000	0.101848000000
H	4.739710000000	-1.314881000000	-2.866784000000
H	5.109148000000	-2.303244000000	1.296831000000
H	5.729182000000	-2.693143000000	-1.066568000000
H	2.340244000000	1.523072000000	-2.197215000000
H	2.741934000000	0.432949000000	2.372528000000
H	2.173364000000	-1.487218000000	3.825742000000
H	1.619196000000	-1.787732000000	2.153932000000
H	3.085563000000	-2.588548000000	2.764638000000
H	4.246317000000	-0.051221000000	4.249616000000
H	5.191182000000	0.583781000000	2.877307000000
H	5.213639000000	-1.155470000000	3.253459000000
H	3.476730000000	1.966182000000	-4.327486000000
H	4.671333000000	1.965381000000	-3.003909000000
H	4.548793000000	0.570843000000	-4.103013000000
H	1.443061000000	0.417230000000	-4.224333000000
H	1.171075000000	-0.599560000000	-2.780726000000
H	2.471742000000	-1.006275000000	-3.924840000000
H	3.662985000000	2.764283000000	0.538117000000
H	1.253407000000	4.099661000000	1.037653000000
H	-3.499239000000	1.847311000000	2.835633000000
H	-4.720985000000	3.083664000000	1.076750000000
H	-3.538474000000	3.904629000000	-0.932166000000
H	0.147145000000	3.759998000000	-1.354935000000
H	-0.300397000000	2.633244000000	-3.507590000000
H	-0.445333000000	1.456825000000	-2.171889000000
H	-1.908942000000	2.125297000000	-2.927507000000
H	-0.843348000000	5.112085000000	-3.094627000000
H	-1.569284000000	5.615224000000	-1.546765000000
H	-2.483929000000	4.592804000000	-2.684275000000
H	0.182586000000	1.065095000000	2.635529000000
H	-0.172236000000	1.749772000000	4.986299000000
H	-0.278112000000	3.165878000000	3.907157000000
H	-1.760040000000	2.439174000000	4.571721000000
H	-0.889573000000	-0.532922000000	4.173737000000
H	-1.471195000000	-0.769353000000	2.508623000000
H	-2.502456000000	0.049572000000	3.714856000000
N	-3.160549000000	-4.591569000000	-0.530056000000
N	-3.463638000000	-3.507515000000	-0.818841000000
N	-3.046531000000	-2.358234000000	-1.003644000000

C	-3.978374000000	-1.220244000000	-0.837423000000
C	-4.303554000000	-1.037383000000	0.646008000000
H	-3.377893000000	-0.861800000000	1.213208000000
H	-4.969491000000	-0.172419000000	0.788101000000
H	-4.799371000000	-1.933550000000	1.049387000000
C	-3.276303000000	0.017639000000	-1.384558000000
H	-3.977623000000	0.864423000000	-1.403692000000
H	-2.420601000000	0.300452000000	-0.752642000000
H	-2.911084000000	-0.171268000000	-2.404428000000
C	-5.251581000000	-1.488451000000	-1.645628000000
H	-5.768708000000	-2.383582000000	-1.271512000000
H	-5.934784000000	-0.630481000000	-1.565735000000
H	-5.004719000000	-1.645111000000	-2.705357000000

2-iso-P
***E* =** -1994.640553
***H* =** -1994.597442
***G* =** -1994.719931
G_{sp} = -1996.527894
N_{imag} = 0
***SP* =** -1997.183342

C	4.224489000000	1.715855000000	1.298004000000
C	3.299353000000	0.667184000000	1.352106000000
C	3.158451000000	-0.123996000000	0.202056000000
C	3.892005000000	0.093854000000	-0.978078000000
C	4.806385000000	1.149721000000	-0.977108000000
C	4.970254000000	1.953394000000	0.149463000000
C	2.516639000000	0.412697000000	2.630390000000
C	3.642108000000	-0.737407000000	-2.227203000000
C	2.469928000000	-0.148039000000	-3.023870000000
C	4.879254000000	-0.879157000000	-3.113617000000
C	3.451017000000	-0.068635000000	3.745951000000
C	1.724530000000	1.650676000000	3.060932000000
N	2.210236000000	-1.204371000000	0.228680000000
C	2.529654000000	-2.532737000000	0.449542000000
C	1.355660000000	-3.216693000000	0.449607000000
N	0.361052000000	-2.277662000000	0.244205000000
C	0.875300000000	-1.035628000000	0.106300000000
C	-1.048543000000	-2.541481000000	0.169795000000
C	-1.630979000000	-2.683350000000	-1.099173000000
C	-3.018674000000	-2.851713000000	-1.150273000000
C	-3.778895000000	-2.880378000000	0.014934000000
C	-3.166893000000	-2.755989000000	1.260277000000
C	-1.783631000000	-2.585771000000	1.366740000000
C	-1.117800000000	-2.414390000000	2.724039000000
C	-1.256655000000	-0.972750000000	3.227722000000
C	-1.651345000000	-3.406764000000	3.759778000000
C	-0.811689000000	-2.671316000000	-2.380585000000
C	-0.968794000000	-3.995513000000	-3.135701000000
C	-1.172223000000	-1.479142000000	-3.271785000000
Au	-0.196740000000	0.718795000000	-0.138773000000
C	-1.207228000000	2.498075000000	-0.318765000000
P	-0.392727000000	4.023038000000	-0.423277000000
H	4.361118000000	2.354570000000	2.172631000000
H	5.394932000000	1.354611000000	-1.871532000000
H	5.685319000000	2.776972000000	0.127534000000
H	1.789278000000	-0.390218000000	2.438747000000
H	3.350226000000	-1.749272000000	-1.906324000000
H	2.259372000000	-0.763786000000	-3.911276000000
H	1.554545000000	-0.088303000000	-2.416454000000
H	2.713851000000	0.871191000000	-3.359808000000

H	4.679990000000	-1.600779000000	-3.918324000000
H	5.750780000000	-1.230040000000	-2.542575000000
H	5.143241000000	0.075145000000	-3.592642000000
H	2.876626000000	-0.290983000000	4.657276000000
H	3.992949000000	-0.977948000000	3.447577000000
H	4.195096000000	0.703164000000	3.995348000000
H	1.119713000000	1.422426000000	3.950866000000
H	1.049041000000	1.990697000000	2.261052000000
H	2.394922000000	2.484671000000	3.317731000000
H	3.555420000000	-2.863356000000	0.581420000000
H	1.136495000000	-4.271990000000	0.579364000000
H	-3.510889000000	-2.956990000000	-2.119467000000
H	-4.861159000000	-3.005213000000	-0.046615000000
H	-3.776903000000	-2.784535000000	2.164582000000
H	-0.043519000000	-2.617354000000	2.599602000000
H	-0.747952000000	-0.856718000000	4.196675000000
H	-0.818834000000	-0.252037000000	2.519508000000
H	-2.317476000000	-0.710795000000	3.363311000000
H	-1.063227000000	-3.334369000000	4.685667000000
H	-1.594631000000	-4.441734000000	3.393556000000
H	-2.698148000000	-3.192965000000	4.021219000000
H	0.250175000000	-2.568823000000	-2.109583000000
H	-0.322409000000	-4.005412000000	-4.025117000000
H	-0.698394000000	-4.851801000000	-2.501210000000
H	-2.006067000000	-4.138258000000	-3.474329000000
H	-0.580834000000	-1.505460000000	-4.198764000000
H	-0.968682000000	-0.525192000000	-2.762724000000
H	-2.237956000000	-1.498034000000	-3.548070000000
N	-1.895923000000	4.839397000000	-0.498561000000
N	-2.893868000000	4.060219000000	-0.450788000000
N	-2.538001000000	2.757741000000	-0.350330000000
C	-3.645354000000	1.758080000000	-0.286350000000
C	-3.524977000000	0.802534000000	-1.474632000000
H	-2.606919000000	0.202738000000	-1.409491000000
H	-4.380389000000	0.110736000000	-1.476866000000
H	-3.518042000000	1.361975000000	-2.421688000000
C	-3.547950000000	0.984884000000	1.030457000000
H	-4.404533000000	0.299976000000	1.117141000000
H	-2.629582000000	0.382752000000	1.072430000000
H	-3.558426000000	1.676356000000	1.885939000000
C	-4.993390000000	2.478114000000	-0.350685000000
H	-5.098245000000	3.047722000000	-1.281810000000
H	-5.786221000000	1.718124000000	-0.303063000000
H	-5.114815000000	3.175610000000	0.486716000000

1-As
***E* =** -3567.415575
***H* =** -3567.379426
***G* =** -3567.487170
G_{sp} = -3569.085287
N_{imag} = 0
***SP* =** -3569.605500

C	-4.284802000000	-0.434738000000	-1.377589000000
C	-2.969044000000	-0.909519000000	-1.338205000000
C	-2.465905000000	-1.303866000000	-0.089557000000
C	-3.216853000000	-1.244406000000	1.097874000000
C	-4.525725000000	-0.765799000000	1.003288000000
C	-5.053969000000	-0.363337000000	-0.221772000000
C	-2.151107000000	-0.988320000000	-2.616908000000
C	-2.608032000000	-1.618795000000	2.440364000000

C	-1.945538000000	-0.392044000000	3.081804000000
C	-3.617861000000	-2.250787000000	3.399016000000
C	-2.774872000000	-1.983330000000	-3.601273000000
C	-1.980520000000	0.394395000000	-3.253431000000
N	-1.112842000000	-1.783314000000	-0.019803000000
C	-0.750563000000	-3.118323000000	-0.015049000000
C	0.606783000000	-3.146462000000	0.023685000000
N	1.024221000000	-1.827656000000	0.023962000000
C	-0.027304000000	-0.978332000000	0.000868000000
C	2.395469000000	-1.402540000000	0.094887000000
C	2.909393000000	-1.017943000000	1.342220000000
C	4.242091000000	-0.593028000000	1.382413000000
C	5.016908000000	-0.559676000000	0.228747000000
C	4.477709000000	-0.951709000000	-0.994914000000
C	3.151825000000	-1.380765000000	-1.090433000000
C	2.533091000000	-1.741871000000	-2.432128000000
C	1.907928000000	-0.498787000000	-3.079644000000
C	3.524277000000	-2.407062000000	-3.387731000000
C	2.086614000000	-1.058949000000	2.619693000000
C	2.662435000000	-2.083832000000	3.602808000000
C	1.979784000000	0.328949000000	3.258580000000
Au	0.023159000000	1.103375000000	-0.002511000000
C	0.081389000000	3.096155000000	-0.006720000000
As	0.143386000000	4.764446000000	-0.010869000000
H	-4.710432000000	-0.115392000000	-2.330949000000
H	-5.141615000000	-0.699599000000	1.900683000000
H	-6.077036000000	0.012026000000	-0.272898000000
H	-1.146732000000	-1.359186000000	-2.363716000000
H	-1.819520000000	-2.364578000000	2.256564000000
H	-1.469021000000	-0.667236000000	4.034844000000
H	-1.180565000000	0.048262000000	2.424508000000
H	-2.698372000000	0.385104000000	3.283456000000
H	-3.099063000000	-2.625340000000	4.292729000000
H	-4.151293000000	-3.090640000000	2.931178000000
H	-4.363263000000	-1.517754000000	3.740845000000
H	-2.153312000000	-2.067490000000	-4.504668000000
H	-2.870057000000	-2.982645000000	-3.152178000000
H	-3.777925000000	-1.654325000000	-3.912537000000
H	-1.343084000000	0.325536000000	-4.147134000000
H	-1.515299000000	1.101466000000	-2.549913000000
H	-2.951139000000	0.810556000000	-3.563439000000
H	-1.487901000000	-3.914982000000	-0.035858000000
H	1.310064000000	-3.973232000000	0.046729000000
H	4.676503000000	-0.282598000000	2.334738000000
H	6.053175000000	-0.222556000000	0.280417000000
H	5.098666000000	-0.915803000000	-1.890487000000
H	1.722430000000	-2.462925000000	-2.245958000000
H	1.425978000000	-0.763754000000	-4.032847000000
H	1.154380000000	-0.033929000000	-2.426199000000
H	2.683445000000	0.255443000000	-3.282184000000
H	2.994908000000	-2.770438000000	-4.279833000000
H	4.033607000000	-3.259744000000	-2.916259000000
H	4.290230000000	-1.697034000000	-3.732714000000
H	1.066280000000	-1.382031000000	2.364937000000
H	2.037954000000	-2.139399000000	4.506417000000
H	2.709674000000	-3.085935000000	3.152324000000
H	3.680341000000	-1.803390000000	3.913564000000
H	1.337959000000	0.288041000000	4.150852000000
H	1.549618000000	1.058392000000	2.555624000000
H	2.967852000000	0.698884000000	3.571907000000

TS-As
E **=** -3888.913537
H **=** -3888.868380
G **=** -3888.997340
G_{sp} **=** -3890.963869
N_{imag} **=** 1, -336 cm^{-1}
SP **=** -3891.610864

C	-1.304377000000	4.449692000000	-1.108814000000
C	-1.918484000000	3.204286000000	-0.943031000000
C	-1.853520000000	2.619233000000	0.330771000000
C	-1.199091000000	3.218260000000	1.419723000000
C	-0.599697000000	4.461870000000	1.201394000000
C	-0.653133000000	5.072669000000	-0.048756000000
C	-2.592242000000	2.521173000000	-2.121417000000
C	-1.081910000000	2.529565000000	2.769712000000
C	0.323588000000	1.941968000000	2.948526000000
C	-1.441986000000	3.463542000000	3.927723000000
C	-3.719814000000	3.382973000000	-2.697122000000
C	-1.561239000000	2.157139000000	-3.194989000000
N	-2.460088000000	1.331769000000	0.523137000000
C	-3.742270000000	1.124176000000	0.998942000000
C	-3.920208000000	-0.221658000000	1.040598000000
N	-2.744976000000	-0.785962000000	0.576540000000
C	-1.834318000000	0.163247000000	0.260852000000
C	-2.489781000000	-2.198010000000	0.497750000000
C	-1.741547000000	-2.799526000000	1.520932000000
C	-1.504865000000	-4.175068000000	1.416865000000
C	-1.995140000000	-4.906206000000	0.341263000000
C	-2.733431000000	-4.278811000000	-0.660399000000
C	-2.994780000000	-2.907865000000	-0.606503000000
C	-3.730892000000	-2.196927000000	-1.731724000000
C	-2.726690000000	-1.648110000000	-2.754825000000
C	-4.773109000000	-3.078982000000	-2.419253000000
C	-1.215168000000	-2.027940000000	2.720382000000
C	-1.943008000000	-2.461078000000	3.997946000000
C	0.302410000000	-2.173965000000	2.863397000000
Au	0.115665000000	-0.103851000000	-0.380653000000
C	2.019192000000	-0.356399000000	-0.962980000000
As	3.386193000000	-0.591532000000	-1.942219000000
H	-1.328813000000	4.933265000000	-2.087409000000
H	-0.073139000000	4.955200000000	2.020168000000
H	-0.174754000000	6.041491000000	-0.199798000000
H	-3.041550000000	1.582155000000	-1.764620000000
H	-1.796791000000	1.693299000000	2.788572000000
H	0.398608000000	1.416329000000	3.912478000000
H	0.574349000000	1.232153000000	2.145425000000
H	1.079867000000	2.742004000000	2.935675000000
H	-1.432783000000	2.907988000000	4.876481000000
H	-2.440557000000	3.904021000000	3.794358000000
H	-0.716582000000	4.285201000000	4.022182000000
H	-4.226377000000	2.850534000000	-3.515211000000
H	-4.467475000000	3.628614000000	-1.929085000000
H	-3.329525000000	4.327520000000	-3.105200000000
H	-2.043743000000	1.614169000000	-4.021002000000
H	-0.764469000000	1.522069000000	-2.778862000000
H	-1.092488000000	3.062007000000	-3.611257000000
H	-4.398971000000	1.947043000000	1.263700000000
H	-4.766138000000	-0.827343000000	1.350994000000
H	-0.926179000000	-4.678715000000	2.193521000000
H	-1.798403000000	-5.977449000000	0.278229000000
H	-3.105432000000	-4.866591000000	-1.499921000000

H	-4.263605000000	-1.338003000000	-1.295344000000
H	-3.251950000000	-1.103472000000	-3.553916000000
H	-1.999611000000	-0.966866000000	-2.288280000000
H	-2.162008000000	-2.474358000000	-3.212855000000
H	-5.367284000000	-2.475810000000	-3.120124000000
H	-5.458107000000	-3.540752000000	-1.693670000000
H	-4.299470000000	-3.881399000000	-3.003898000000
H	-1.425455000000	-0.959751000000	2.563953000000
H	-1.589834000000	-1.873032000000	4.857747000000
H	-3.030046000000	-2.321017000000	3.905823000000
H	-1.755569000000	-3.523789000000	4.215366000000
H	0.663518000000	-1.561029000000	3.702391000000
H	0.824394000000	-1.843373000000	1.953011000000
H	0.585508000000	-3.218131000000	3.066021000000
N	4.858857000000	-0.650075000000	0.008069000000
N	4.056301000000	-0.353628000000	0.911503000000
N	2.956345000000	-0.187874000000	1.204736000000
C	6.272362000000	-0.236622000000	0.117881000000
C	6.955181000000	-0.754430000000	-1.144919000000
H	6.837919000000	-1.844444000000	-1.223239000000
H	6.516174000000	-0.293548000000	-2.040889000000
H	8.026826000000	-0.512887000000	-1.114511000000
C	6.362624000000	1.289136000000	0.190915000000
H	5.883404000000	1.739655000000	-0.690289000000
H	5.854567000000	1.664172000000	1.092250000000
H	7.413577000000	1.611377000000	0.226555000000
C	6.896000000000	-0.879752000000	1.358427000000
H	6.391310000000	-0.528252000000	2.270377000000
H	6.804494000000	-1.973723000000	1.307698000000
H	7.961629000000	-0.616856000000	1.427695000000

2-As
***E* =** -3889.014038
***H* =** -3888.970109
***G* =** -3889.095115
G_{sp} = -3891.059579
N_{imag} = 0
***SP* =** -3891.712402

C	1.514421000000	-4.333887000000	-1.156439000000
C	1.961316000000	-3.008310000000	-1.166542000000
C	2.250020000000	-2.417285000000	0.072781000000
C	2.109401000000	-3.091425000000	1.297833000000
C	1.662338000000	-4.414650000000	1.253220000000
C	1.367375000000	-5.029869000000	0.038872000000
C	2.095547000000	-2.258267000000	-2.481468000000
C	2.359596000000	-2.396288000000	2.626817000000
C	1.036198000000	-1.891324000000	3.216228000000
C	3.100371000000	-3.286485000000	3.626580000000
C	3.065409000000	-2.966526000000	-3.432103000000
C	0.723149000000	-2.057168000000	-3.132707000000
N	2.691818000000	-1.050990000000	0.092803000000
C	4.014601000000	-0.650617000000	0.167410000000
C	4.002550000000	0.707367000000	0.164991000000
N	2.674781000000	1.086034000000	0.073324000000
C	1.856834000000	0.009449000000	0.034039000000
C	2.209998000000	2.446588000000	0.061663000000
C	1.667640000000	2.981896000000	1.239895000000
C	1.233939000000	4.312200000000	1.197447000000
C	1.337399000000	5.062739000000	0.032531000000
C	1.868355000000	4.497864000000	-1.125648000000

C	2.311477000000	3.173654000000	-1.138706000000
C	2.802628000000	2.514559000000	-2.418816000000
C	1.644262000000	1.774422000000	-3.102836000000
C	3.462788000000	3.494072000000	-3.388653000000
C	1.560024000000	2.191455000000	2.533833000000
C	2.564865000000	2.717255000000	3.565436000000
C	0.132445000000	2.201537000000	3.090008000000
Au	-0.225071000000	0.040008000000	0.034149000000
C	-2.261754000000	0.125801000000	0.119366000000
As	-3.467137000000	-0.690907000000	-1.006583000000
H	1.273287000000	-4.825298000000	-2.101193000000
H	1.532796000000	-4.970186000000	2.183152000000
H	1.014231000000	-6.062041000000	0.026107000000
H	2.509285000000	-1.261038000000	-2.269022000000
H	2.994379000000	-1.517812000000	2.436501000000
H	1.216569000000	-1.348760000000	4.156469000000
H	0.514333000000	-1.217406000000	2.519465000000
H	0.364677000000	-2.737032000000	3.429896000000
H	3.357038000000	-2.707353000000	4.525037000000
H	4.029050000000	-3.689886000000	3.198027000000
H	2.477714000000	-4.132799000000	3.952178000000
H	3.190847000000	-2.379774000000	-4.353747000000
H	4.053897000000	-3.098716000000	-2.969055000000
H	2.688653000000	-3.960097000000	-3.718103000000
H	0.820474000000	-1.474581000000	-4.060651000000
H	0.040674000000	-1.520867000000	-2.455548000000
H	0.263123000000	-3.024954000000	-3.385101000000
H	4.830882000000	-1.364349000000	0.220585000000
H	4.806999000000	1.434861000000	0.215460000000
H	0.806639000000	4.763319000000	2.094792000000
H	0.993417000000	6.098019000000	0.021438000000
H	1.930159000000	5.097155000000	-2.034227000000
H	3.559869000000	1.764364000000	-2.144287000000
H	1.999515000000	1.254655000000	-4.005620000000
H	1.180964000000	1.034292000000	-2.433149000000
H	0.861874000000	2.489220000000	-3.400093000000
H	3.916650000000	2.942071000000	-4.223744000000
H	4.248864000000	4.085437000000	-2.897483000000
H	2.728580000000	4.189725000000	-3.821121000000
H	1.819843000000	1.144188000000	2.321814000000
H	2.512456000000	2.121966000000	4.488984000000
H	3.594601000000	2.670705000000	3.181435000000
H	2.347002000000	3.764711000000	3.824974000000
H	0.073534000000	1.552692000000	3.976493000000
H	-0.603799000000	1.842442000000	2.353493000000
H	-0.165032000000	3.213997000000	3.403214000000
N	-4.709636000000	0.145626000000	0.094016000000
N	-4.182705000000	0.849398000000	1.056925000000
N	-2.887803000000	0.852999000000	1.087757000000
C	-6.192244000000	0.123369000000	0.037196000000
C	-6.629408000000	-0.731120000000	-1.149003000000
H	-6.252007000000	-0.318837000000	-2.096251000000
H	-6.271408000000	-1.766053000000	-1.046947000000
H	-7.727304000000	-0.749714000000	-1.194561000000
C	-6.715922000000	-0.480862000000	1.341995000000
H	-6.337230000000	-1.506019000000	1.464963000000
H	-6.383335000000	0.120936000000	2.197202000000
H	-7.814880000000	-0.510082000000	1.327518000000
C	-6.692089000000	1.559272000000	-0.137605000000
H	-6.365347000000	2.180503000000	0.705925000000
H	-6.292463000000	1.990193000000	-1.067139000000

H	-7.790347000000	1.569506000000	-0.188052000000

TS-iso-As
E **=** -3888.911129
H **=** -3888.866232
G **=** -3888.993024
G_{sp} **=** -3890.961278
N_{imag} **=** 1, -339 cm^{-1}
SP **=** -3891.609047

C	4.402677000000	-1.392511000000	-1.843262000000
C	3.615942000000	-0.281170000000	-1.526952000000
C	3.287309000000	-0.088969000000	-0.175728000000
C	3.691582000000	-0.963207000000	0.844955000000
C	4.478965000000	-2.058326000000	0.476902000000
C	4.834021000000	-2.269562000000	-0.852003000000
C	3.097082000000	0.630749000000	-2.626422000000
C	3.275081000000	-0.772776000000	2.293572000000
C	2.366980000000	-1.919304000000	2.751757000000
C	4.493354000000	-0.622127000000	3.209333000000
C	4.225639000000	1.150821000000	-3.520434000000
C	2.023533000000	-0.093486000000	-3.447243000000
N	2.475625000000	1.043834000000	0.173589000000
C	2.968885000000	2.280109000000	0.547978000000
C	1.887243000000	3.063305000000	0.797374000000
N	0.773375000000	2.278629000000	0.556778000000
C	1.125341000000	1.026571000000	0.181947000000
C	-0.588386000000	2.709267000000	0.714931000000
C	-1.285854000000	2.319077000000	1.868617000000
C	-2.621299000000	2.725394000000	1.976128000000
C	-3.215908000000	3.501069000000	0.987410000000
C	-2.490303000000	3.887757000000	-0.137821000000
C	-1.161476000000	3.491734000000	-0.304523000000
C	-0.386251000000	3.829117000000	-1.569067000000
C	-0.509264000000	2.686275000000	-2.585950000000
C	-0.808515000000	5.156831000000	-2.197717000000
C	-0.631803000000	1.549220000000	3.005316000000
C	-0.470570000000	2.461366000000	4.227674000000
C	-1.396046000000	0.272026000000	3.361273000000
Au	-0.102379000000	-0.622028000000	-0.085036000000
C	-1.209319000000	-2.298117000000	-0.187816000000
As	-1.343780000000	-3.978918000000	0.079483000000
H	4.671691000000	-1.580640000000	-2.884355000000
H	4.807996000000	-2.763260000000	1.243008000000
H	5.443744000000	-3.133591000000	-1.120002000000
H	2.620586000000	1.503533000000	-2.154127000000
H	2.691893000000	0.157435000000	2.362741000000
H	2.026004000000	-1.746680000000	3.783425000000
H	1.482636000000	-2.011905000000	2.102843000000
H	2.905163000000	-2.879169000000	2.729659000000
H	4.172192000000	-0.438998000000	4.244960000000
H	5.131278000000	0.215365000000	2.891466000000
H	5.106864000000	-1.535673000000	3.206381000000
H	3.825538000000	1.855063000000	-4.264167000000
H	4.996246000000	1.669680000000	-2.932378000000
H	4.711540000000	0.330911000000	-4.070050000000
H	1.607984000000	0.576110000000	-4.214699000000
H	1.200465000000	-0.442229000000	-2.804741000000
H	2.450437000000	-0.972245000000	-3.954468000000
H	4.033915000000	2.483342000000	0.601242000000
H	1.805396000000	4.097742000000	1.116890000000

H	-3.198739000000	2.437036000000	2.856816000000
H	-4.256531000000	3.811882000000	1.094124000000
H	-2.972075000000	4.496966000000	-0.903173000000
H	0.676686000000	3.918320000000	-1.298465000000
H	0.099863000000	2.899191000000	-3.477395000000
H	-0.181827000000	1.724034000000	-2.163076000000
H	-1.557124000000	2.572950000000	-2.903314000000
H	-0.124420000000	5.413741000000	-3.018635000000
H	-0.792874000000	5.976798000000	-1.465492000000
H	-1.820001000000	5.097358000000	-2.625962000000
H	0.374654000000	1.246304000000	2.682240000000
H	0.051372000000	1.929259000000	5.036262000000
H	0.105827000000	3.364283000000	3.978481000000
H	-1.451996000000	2.780956000000	4.610224000000
H	-0.889170000000	-0.251534000000	4.184920000000
H	-1.445602000000	-0.414528000000	2.502729000000
H	-2.423115000000	0.493599000000	3.689093000000
N	-3.708577000000	-3.985957000000	-0.713574000000
N	-3.799159000000	-2.860655000000	-0.971677000000
N	-3.223903000000	-1.776411000000	-1.110498000000
C	-3.996248000000	-0.528994000000	-0.893493000000
C	-4.235738000000	-0.335565000000	0.604573000000
H	-3.272819000000	-0.283476000000	1.132965000000
H	-4.786481000000	0.600099000000	0.785943000000
H	-4.818445000000	-1.173372000000	1.017284000000
C	-3.166514000000	0.619975000000	-1.454972000000
H	-3.751561000000	1.550684000000	-1.422352000000
H	-2.251499000000	0.775982000000	-0.863885000000
H	-2.879633000000	0.410332000000	-2.495699000000
C	-5.324478000000	-0.619374000000	-1.650593000000
H	-5.940939000000	-1.442563000000	-1.261935000000
H	-5.884551000000	0.319919000000	-1.536358000000
H	-5.143417000000	-0.793629000000	-2.720864000000

2-As-iso
***E* =** -3889.016794
***H* =** -3888.973237
***G* =** -3889.094892
G_{sp} = -3891.062203
N_{imag} = 0
***SP* =** -3891.716576

C	4.161706000000	1.606993000000	1.407023000000
C	3.290358000000	0.511848000000	1.402664000000
C	3.172230000000	-0.209303000000	0.204812000000
C	3.878458000000	0.119705000000	-0.965902000000
C	4.738018000000	1.219036000000	-0.906662000000
C	4.876838000000	1.956531000000	0.267515000000
C	2.538224000000	0.136066000000	2.669265000000
C	3.656804000000	-0.643168000000	-2.262796000000
C	2.461490000000	-0.051859000000	-3.022608000000
C	4.897026000000	-0.682966000000	-3.155606000000
C	3.509618000000	-0.375078000000	3.739042000000
C	1.696129000000	1.303658000000	3.191234000000
N	2.271144000000	-1.329065000000	0.169251000000
C	2.644745000000	-2.654122000000	0.307992000000
C	1.498596000000	-3.383306000000	0.273747000000
N	0.465777000000	-2.474605000000	0.130437000000
C	0.929783000000	-1.207207000000	0.063550000000
C	-0.933194000000	-2.788580000000	0.044333000000
C	-1.517277000000	-2.873323000000	-1.228973000000

C	-2.898237000000	-3.089324000000	-1.285923000000
C	-3.650600000000	-3.216663000000	-0.122436000000
C	-3.036461000000	-3.147604000000	1.126264000000
C	-1.659697000000	-2.934739000000	1.238605000000
C	-0.992246000000	-2.825148000000	2.601422000000
C	-1.162400000000	-1.417827000000	3.186091000000
C	-1.497977000000	-3.885345000000	3.582411000000
C	-0.706679000000	-2.753043000000	-2.510185000000
C	-0.807611000000	-4.040527000000	-3.335504000000
C	-1.125840000000	-1.532002000000	-3.333938000000
Au	-0.206775000000	0.516070000000	-0.087547000000
C	-1.272223000000	2.269061000000	-0.185073000000
As	-0.420098000000	3.906475000000	-0.253607000000
H	4.278797000000	2.194572000000	2.319546000000
H	5.302913000000	1.510774000000	-1.792324000000
H	5.548767000000	2.815620000000	0.290745000000
H	1.845903000000	-0.685427000000	2.431768000000
H	3.405191000000	-1.683136000000	-2.003243000000
H	2.273840000000	-0.619124000000	-3.946669000000
H	1.545325000000	-0.065640000000	-2.413612000000
H	2.664703000000	0.995116000000	-3.294339000000
H	4.724004000000	-1.360983000000	-4.003175000000
H	5.782328000000	-1.034555000000	-2.606614000000
H	5.123464000000	0.308297000000	-3.575047000000
H	2.959298000000	-0.684175000000	4.639837000000
H	4.087768000000	-1.236313000000	3.373605000000
H	4.221627000000	0.411819000000	4.031141000000
H	1.117273000000	0.989022000000	4.071956000000
H	0.992682000000	1.662096000000	2.424167000000
H	2.330457000000	2.150432000000	3.493717000000
H	3.683734000000	-2.950988000000	0.414007000000
H	1.322857000000	-4.452486000000	0.340980000000
H	-3.391366000000	-3.152522000000	-2.258285000000
H	-4.728011000000	-3.376452000000	-0.187880000000
H	-3.639812000000	-3.255008000000	2.029046000000
H	0.085856000000	-2.996154000000	2.462288000000
H	-0.650545000000	-1.343850000000	4.157492000000
H	-0.747281000000	-0.647685000000	2.517543000000
H	-2.228526000000	-1.190579000000	3.341236000000
H	-0.904950000000	-3.852303000000	4.507376000000
H	-1.422408000000	-4.896801000000	3.158461000000
H	-2.547006000000	-3.708497000000	3.861863000000
H	0.350782000000	-2.616990000000	-2.237232000000
H	-0.167050000000	-3.973098000000	-4.226671000000
H	-0.494430000000	-4.916278000000	-2.749050000000
H	-1.839573000000	-4.212306000000	-3.676850000000
H	-0.538495000000	-1.480599000000	-4.262404000000
H	-0.963653000000	-0.598751000000	-2.774155000000
H	-2.190813000000	-1.584341000000	-3.608575000000
N	-2.119238000000	4.649763000000	-0.267982000000
N	-3.034329000000	3.793248000000	-0.228034000000
N	-2.602031000000	2.492612000000	-0.182228000000
C	-3.673696000000	1.449476000000	-0.140946000000
C	-3.543293000000	0.549216000000	-1.371010000000
H	-2.605954000000	-0.023225000000	-1.347632000000
H	-4.376095000000	-0.169389000000	-1.389346000000
H	-3.571193000000	1.148388000000	-2.293086000000
C	-3.526428000000	0.625526000000	1.140143000000
H	-4.358496000000	-0.090582000000	1.212316000000
H	-2.588711000000	0.052487000000	1.141771000000
H	-3.544094000000	1.279674000000	2.024407000000

C	-5.050665000000	2.116903000000	-0.151995000000
H	-5.197900000000	2.717500000000	-1.057496000000
H	-5.810427000000	1.322899000000	-0.120236000000
H	-5.184564000000	2.775341000000	0.714432000000

1-Sb

***E* =** -1571.935912

***H* =** -1571.899633

***G* =** -1572.007715

G_{sp} = -1573.339761

N_{imag} = 0

***SP* =** -1573.858974

C	-4.175197000000	1.184073000000	-1.382749000000
C	-3.191055000000	0.190336000000	-1.337505000000
C	-2.905262000000	-0.373999000000	-0.085442000000
C	-3.557674000000	0.009334000000	1.099806000000
C	-4.534407000000	1.002880000000	0.999296000000
C	-4.838858000000	1.585894000000	-0.229189000000
C	-2.487738000000	-0.241438000000	-2.613932000000
C	-3.169092000000	-0.582320000000	2.446030000000
C	-2.043901000000	0.243913000000	3.083670000000
C	-4.353174000000	-0.713197000000	3.404792000000
C	-3.478656000000	-0.886601000000	-3.588632000000
C	-1.746815000000	0.930528000000	-3.264537000000
N	-1.887847000000	-1.386041000000	-0.010230000000
C	-2.131527000000	-2.747707000000	0.003359000000
C	-0.916527000000	-3.353325000000	0.043289000000
N	0.024791000000	-2.339598000000	0.034656000000
C	-0.562031000000	-1.121906000000	0.005708000000
C	1.445913000000	-2.542039000000	0.103291000000
C	2.077774000000	-2.406912000000	1.348390000000
C	3.463842000000	-2.595252000000	1.387278000000
C	4.175647000000	-2.905499000000	0.234224000000
C	3.517989000000	-3.035541000000	-0.987425000000
C	2.136149000000	-2.854450000000	-1.081425000000
C	1.419870000000	-2.923564000000	-2.421415000000
C	1.388015000000	-1.536706000000	-3.078021000000
C	2.027500000000	-3.956693000000	-3.370988000000
C	1.318584000000	-2.082590000000	2.624606000000
C	1.394220000000	-3.251979000000	3.612082000000
C	1.822483000000	-0.782453000000	3.258013000000
Au	0.370960000000	0.745525000000	-0.006774000000
C	1.260646000000	2.520659000000	-0.015116000000
Sb	2.104046000000	4.193357000000	-0.021574000000
H	-4.422415000000	1.649597000000	-2.338897000000
H	-5.062153000000	1.332620000000	1.894704000000
H	-5.601507000000	2.364016000000	-0.284802000000
H	-1.735658000000	-1.001599000000	-2.355130000000
H	-2.777631000000	-1.595668000000	2.268190000000
H	-1.736420000000	-0.201576000000	4.041861000000
H	-1.161411000000	0.304824000000	2.429034000000
H	-2.387791000000	1.271808000000	3.274851000000
H	-4.046618000000	-1.269909000000	4.301600000000
H	-5.195917000000	-1.244143000000	2.939292000000
H	-4.710738000000	0.271071000000	3.741331000000
H	-2.955050000000	-1.235799000000	-4.490574000000
H	-3.987991000000	-1.746228000000	-3.129282000000
H	-4.248040000000	-0.165500000000	-3.903950000000
H	-1.201017000000	0.587245000000	-4.155629000000
H	-1.023811000000	1.379792000000	-2.566937000000

H	-2.448833000000	1.716495000000	-3.582031000000
H	-3.138888000000	-3.152393000000	-0.012552000000
H	-0.634051000000	-4.401224000000	0.072450000000
H	3.991261000000	-2.495298000000	2.337967000000
H	5.256346000000	-3.045934000000	0.284716000000
H	4.092590000000	-3.275200000000	-1.882589000000
H	0.378450000000	-3.225393000000	-2.231276000000
H	0.839904000000	-1.576069000000	-4.031530000000
H	0.905583000000	-0.789563000000	-2.429963000000
H	2.412021000000	-1.188537000000	-3.281873000000
H	1.391364000000	-4.064365000000	-4.260857000000
H	2.122567000000	-4.941956000000	-2.892427000000
H	3.023359000000	-3.646977000000	-3.720638000000
H	0.258964000000	-1.933356000000	2.369073000000
H	0.807211000000	-3.028584000000	4.514938000000
H	1.003168000000	-4.177525000000	3.164970000000
H	2.433360000000	-3.438300000000	3.923290000000
H	1.224540000000	-0.537129000000	4.147981000000
H	1.751512000000	0.057492000000	2.550425000000
H	2.872672000000	-0.875612000000	3.573953000000

TS-Sb
***E* =** -1893.442696
***H* =** -1893.397191
***G* =** -1893.529421
G_{sp} = -1895.228661
N_{imag} = 1, -257 cm^{-1}
***SP* =** -1895.87329690

C	-1.797063000000	4.326619000000	-1.271197000000
C	-2.305064000000	3.041477000000	-1.054291000000
C	-2.247835000000	2.539567000000	0.254530000000
C	-1.705444000000	3.260490000000	1.332071000000
C	-1.211485000000	4.539568000000	1.062526000000
C	-1.257435000000	5.067963000000	-0.225412000000
C	-2.875184000000	2.241456000000	-2.213626000000
C	-1.589505000000	2.655207000000	2.722093000000
C	-0.185199000000	2.073861000000	2.931478000000
C	-1.941296000000	3.651287000000	3.828912000000
C	-4.086174000000	2.949426000000	-2.829429000000
C	-1.797690000000	1.959013000000	-3.265489000000
N	-2.746835000000	1.215716000000	0.501470000000
C	-4.014191000000	0.924971000000	0.974927000000
C	-4.085012000000	-0.427779000000	1.069909000000
N	-2.863867000000	-0.914055000000	0.636236000000
C	-2.027960000000	0.091013000000	0.287711000000
C	-2.498364000000	-2.303282000000	0.612654000000
C	-1.707578000000	-2.804239000000	1.657924000000
C	-1.368079000000	-4.161204000000	1.609950000000
C	-1.800025000000	-4.970675000000	0.565754000000
C	-2.579549000000	-4.441506000000	-0.460948000000
C	-2.943172000000	-3.092989000000	-0.462832000000
C	-3.722028000000	-2.484987000000	-1.619168000000
C	-2.752381000000	-1.918016000000	-2.665516000000
C	-4.700946000000	-3.465008000000	-2.265908000000
C	-1.240115000000	-1.945132000000	2.821834000000
C	-1.943319000000	-2.367425000000	4.116650000000
C	0.283315000000	-1.981443000000	2.973744000000
Au	-0.058824000000	-0.058368000000	-0.351747000000
C	1.846876000000	-0.196097000000	-0.919951000000
Sb	3.448985000000	-0.424638000000	-1.912348000000
H	-1.819900000000	4.748565000000	-2.277983000000

H	-0.774343000000	5.128629000000	1.870021000000
H	-0.861834000000	6.066744000000	-0.415734000000
H	-3.219796000000	1.271044000000	-1.826246000000
H	-2.306680000000	1.823179000000	2.790320000000
H	-0.109613000000	1.603119000000	3.923576000000
H	0.063734000000	1.320759000000	2.168528000000
H	0.572802000000	2.869892000000	2.869833000000
H	-1.955705000000	3.139937000000	4.802043000000
H	-2.927652000000	4.108130000000	3.663537000000
H	-1.197205000000	4.458526000000	3.897236000000
H	-4.515226000000	2.338714000000	-3.637193000000
H	-4.868914000000	3.128820000000	-2.078099000000
H	-3.800080000000	3.921147000000	-3.259765000000
H	-2.206706000000	1.331957000000	-4.071423000000
H	-0.936576000000	1.436879000000	-2.821307000000
H	-1.433612000000	2.894694000000	-3.716893000000
H	-4.736591000000	1.702722000000	1.202378000000
H	-4.883131000000	-1.086379000000	1.398626000000
H	-0.753995000000	-4.587514000000	2.405515000000
H	-1.523505000000	-6.025896000000	0.546614000000
H	-2.902636000000	-5.088697000000	-1.276700000000
H	-4.312178000000	-1.644325000000	-1.223309000000
H	-3.309262000000	-1.439448000000	-3.485307000000
H	-2.067818000000	-1.175946000000	-2.227958000000
H	-2.138168000000	-2.726438000000	-3.090523000000
H	-5.329952000000	-2.935849000000	-2.995626000000
H	-5.357750000000	-3.935919000000	-1.520381000000
H	-4.173083000000	-4.261382000000	-2.811059000000
H	-1.520164000000	-0.901525000000	2.616231000000
H	-1.633694000000	-1.719199000000	4.949629000000
H	-3.036770000000	-2.303497000000	4.016357000000
H	-1.687709000000	-3.404789000000	4.382047000000
H	0.598254000000	-1.303232000000	3.780607000000
H	0.785441000000	-1.663097000000	2.047821000000
H	0.635083000000	-2.992442000000	3.230260000000
N	4.767451000000	0.085842000000	0.268727000000
N	3.866044000000	0.235031000000	1.110105000000
N	2.756505000000	0.294723000000	1.384428000000
C	6.176120000000	0.000813000000	0.704893000000
C	6.994411000000	-0.160406000000	-0.572648000000
H	6.708271000000	-1.080726000000	-1.101818000000
H	6.828496000000	0.692774000000	-1.245885000000
H	8.064030000000	-0.214496000000	-0.326673000000
C	6.569081000000	1.289768000000	1.429929000000
H	6.414343000000	2.158647000000	0.774945000000
H	5.961149000000	1.423082000000	2.337262000000
H	7.627747000000	1.254062000000	1.725412000000
C	6.363178000000	-1.213985000000	1.616483000000
H	5.754339000000	-1.111288000000	2.527398000000
H	6.057686000000	-2.132073000000	1.094865000000
H	7.417316000000	-1.311009000000	1.914506000000

2-Sb
E **=** -1893.545285
H **=** -1893.501070
G **=** -1893.627913
G_{sp} **=** -1895.326251
N_{imag} **=** 0
SP **=** -1895.977046

C	-1.486040000000	4.427202000000	-0.894251000000

C	-2.030753000000	3.141271000000	-0.967765000000
C	-2.273153000000	2.474494000000	0.243086000000
C	-1.989998000000	3.035639000000	1.499174000000
C	-1.446326000000	4.323254000000	1.518543000000
C	-1.197359000000	5.012655000000	0.334830000000
C	-2.301528000000	2.499334000000	-2.318331000000
C	-2.200688000000	2.267183000000	2.793773000000
C	-0.857321000000	1.777843000000	3.348225000000
C	-2.963181000000	3.090385000000	3.834941000000
C	-3.220726000000	3.366792000000	-3.182833000000
C	-0.984366000000	2.192258000000	-3.039448000000
N	-2.805789000000	1.141924000000	0.197389000000
C	-4.146193000000	0.819398000000	0.316535000000
C	-4.218089000000	-0.535672000000	0.257177000000
N	-2.921222000000	-0.989413000000	0.091356000000
C	-2.040799000000	0.037122000000	0.060664000000
C	-2.540546000000	-2.373782000000	0.009462000000
C	-1.980326000000	-2.985257000000	1.141584000000
C	-1.633362000000	-4.336992000000	1.032182000000
C	-1.835719000000	-5.035412000000	-0.151938000000
C	-2.380972000000	-4.395888000000	-1.263668000000
C	-2.739666000000	-3.047397000000	-1.209824000000
C	-3.245833000000	-2.311080000000	-2.441133000000
C	-2.080292000000	-1.594533000000	-3.138036000000
C	-3.985843000000	-3.215767000000	-3.425733000000
C	-1.771671000000	-2.254685000000	2.458344000000
C	-2.796493000000	-2.727411000000	3.496112000000
C	-0.338655000000	-2.409174000000	2.977415000000
Au	0.038188000000	-0.106708000000	0.016856000000
C	2.067567000000	-0.286235000000	0.069297000000
Sb	3.438093000000	0.852673000000	-0.949007000000
H	-1.277110000000	4.974738000000	-1.815414000000
H	-1.203494000000	4.789176000000	2.475204000000
H	-0.767695000000	6.014837000000	0.370905000000
H	-2.815137000000	1.541464000000	-2.146041000000
H	-2.809313000000	1.378735000000	2.568819000000
H	-1.012883000000	1.185938000000	4.262691000000
H	-0.324456000000	1.153048000000	2.614777000000
H	-0.208405000000	2.631103000000	3.598876000000
H	-3.169236000000	2.476478000000	4.723474000000
H	-3.921098000000	3.454610000000	3.436582000000
H	-2.378381000000	3.961404000000	4.166036000000
H	-3.450388000000	2.852146000000	-4.127082000000
H	-4.167683000000	3.582994000000	-2.667706000000
H	-2.744337000000	4.326013000000	-3.435108000000
H	-1.178634000000	1.688770000000	-3.997928000000
H	-0.343859000000	1.538638000000	-2.427462000000
H	-0.427937000000	3.119308000000	-3.247433000000
H	-4.913505000000	1.577858000000	0.436738000000
H	-5.063635000000	-1.214556000000	0.313043000000
H	-1.195779000000	-4.846923000000	1.892131000000
H	-1.558420000000	-6.088680000000	-0.215104000000
H	-2.520694000000	-4.955687000000	-2.188563000000
H	-3.956920000000	-1.540544000000	-2.105858000000
H	-2.443947000000	-1.022592000000	-4.005146000000
H	-1.559960000000	-0.903941000000	-2.457652000000
H	-1.342829000000	-2.330212000000	-3.493307000000
H	-4.443485000000	-2.607238000000	-4.218391000000
H	-4.780277000000	-3.792554000000	-2.930688000000
H	-3.300826000000	-3.923696000000	-3.915288000000
H	-1.941704000000	-1.181669000000	2.288752000000

H	-2.669406000000	-2.175827000000	4.439395000000
H	-3.826287000000	-2.573453000000	3.141169000000
H	-2.668703000000	-3.799752000000	3.710211000000
H	-0.201582000000	-1.793352000000	3.878688000000
H	0.409291000000	-2.096793000000	2.231553000000
H	-0.126542000000	-3.452241000000	3.257759000000
N	4.616798000000	-0.491061000000	0.138423000000
N	3.947121000000	-1.319450000000	0.870280000000
N	2.645178000000	-1.231325000000	0.848053000000
C	6.091501000000	-0.641083000000	0.208686000000
C	6.731761000000	0.395368000000	-0.710185000000
H	6.414986000000	0.248861000000	-1.753961000000
H	6.469506000000	1.417677000000	-0.397939000000
H	7.825631000000	0.296812000000	-0.668908000000
C	6.539373000000	-0.407681000000	1.653717000000
H	6.253003000000	0.602211000000	1.982170000000
H	6.064518000000	-1.140406000000	2.318552000000
H	7.631810000000	-0.506503000000	1.730049000000
C	6.463429000000	-2.051913000000	-0.254289000000
H	5.993363000000	-2.799033000000	0.397694000000
H	6.117045000000	-2.217403000000	-1.285013000000
H	7.554799000000	-2.182282000000	-0.223895000000

TS-iso-Sb
***E* =** -1893.437318
***H* =** -1893.392456
***G* =** -1893.518081
G_{sp} = -1895.22116
N_{imag} = 1, -305 cm^{-1}
***SP* =** -1895.869146

C	-3.419562000000	-3.538540000000	0.312786000000
C	-3.264822000000	-2.187017000000	0.633557000000
C	-2.993387000000	-1.301881000000	-0.422434000000
C	-2.865488000000	-1.714741000000	-1.757671000000
C	-3.029630000000	-3.077288000000	-2.025620000000
C	-3.304491000000	-3.979553000000	-1.002827000000
C	-3.319770000000	-1.723336000000	2.080144000000
C	-2.536653000000	-0.750109000000	-2.885214000000
C	-1.172847000000	-1.078504000000	-3.501988000000
C	-3.640856000000	-0.739322000000	-3.946896000000
C	-4.537885000000	-2.278522000000	2.821950000000
C	-2.016825000000	-2.088229000000	2.802451000000
N	-2.802885000000	0.089087000000	-0.117289000000
C	-3.819272000000	1.017307000000	0.013280000000
C	-3.218935000000	2.202601000000	0.293986000000
N	-1.856465000000	1.957567000000	0.310117000000
C	-1.588787000000	0.654256000000	0.065045000000
C	-0.861593000000	2.959082000000	0.579065000000
C	-0.524452000000	3.853847000000	-0.453947000000
C	0.410331000000	4.848937000000	-0.161816000000
C	0.994788000000	4.932813000000	1.100701000000
C	0.659622000000	4.018536000000	2.091925000000
C	-0.280661000000	3.008434000000	1.854869000000
C	-0.639448000000	2.034973000000	2.965254000000
C	0.602376000000	1.305664000000	3.487286000000
C	-1.384815000000	2.753423000000	4.095191000000
C	-1.086407000000	3.683712000000	-1.857002000000
C	-1.185941000000	4.996294000000	-2.633371000000
C	-0.242894000000	2.659840000000	-2.629847000000
Au	0.229343000000	-0.349987000000	0.067230000000

C	1.903401000000	-1.445629000000	0.124887000000
Sb	2.750247000000	-3.144861000000	0.348932000000
H	-3.620299000000	-4.259456000000	1.107023000000
H	-2.930572000000	-3.437393000000	-3.051601000000
H	-3.422077000000	-5.039765000000	-1.231424000000
H	-3.405122000000	-0.626335000000	2.084197000000
H	-2.472945000000	0.264469000000	-2.463795000000
H	-0.917236000000	-0.342981000000	-4.279140000000
H	-0.379731000000	-1.070961000000	-2.738485000000
H	-1.182625000000	-2.075172000000	-3.969016000000
H	-3.409853000000	-0.002117000000	-4.729667000000
H	-4.615626000000	-0.483347000000	-3.507127000000
H	-3.734914000000	-1.722938000000	-4.431331000000
H	-4.589849000000	-1.850906000000	3.833506000000
H	-5.473251000000	-2.038270000000	2.296280000000
H	-4.479022000000	-3.371519000000	2.931249000000
H	-2.034861000000	-1.716760000000	3.838127000000
H	-1.140678000000	-1.660077000000	2.291386000000
H	-1.884922000000	-3.180755000000	2.831437000000
H	-4.864653000000	0.747393000000	-0.099719000000
H	-3.627529000000	3.191565000000	0.478008000000
H	0.695866000000	5.564807000000	-0.933128000000
H	1.725967000000	5.715575000000	1.308479000000
H	1.132210000000	4.088418000000	3.073576000000
H	-1.315372000000	1.272489000000	2.552070000000
H	0.311829000000	0.549826000000	4.231529000000
H	1.137679000000	0.795618000000	2.671645000000
H	1.300047000000	2.003579000000	3.974659000000
H	-1.673475000000	2.037375000000	4.878519000000
H	-2.295597000000	3.245011000000	3.723180000000
H	-0.748952000000	3.523143000000	4.558702000000
H	-2.104837000000	3.275901000000	-1.768637000000
H	-1.722620000000	4.830245000000	-3.577979000000
H	-1.722755000000	5.767013000000	-2.061905000000
H	-0.191245000000	5.389266000000	-2.890672000000
H	-0.661470000000	2.493530000000	-3.634013000000
H	-0.197723000000	1.691395000000	-2.107841000000
H	0.789198000000	3.026884000000	-2.742333000000
N	5.077669000000	-1.838820000000	0.044461000000
N	4.647732000000	-0.772447000000	-0.074336000000
N	3.711873000000	0.022996000000	-0.013372000000
C	3.705086000000	1.198505000000	-0.922387000000
C	3.169555000000	0.769562000000	-2.290355000000
H	2.146687000000	0.378531000000	-2.192231000000
H	3.164368000000	1.626163000000	-2.981422000000
H	3.800955000000	-0.022608000000	-2.720749000000
C	2.802325000000	2.250042000000	-0.286567000000
H	2.779186000000	3.152022000000	-0.915486000000
H	1.771623000000	1.879066000000	-0.177366000000
H	3.176773000000	2.525618000000	0.709984000000
C	5.125353000000	1.754915000000	-1.053910000000
H	5.793772000000	1.020262000000	-1.525656000000
H	5.112576000000	2.661985000000	-1.674999000000
H	5.530422000000	2.011914000000	-0.064701000000

2-Sb-iso

***E* =** -1893.546199

***H* =** -1893.502202

***G* =** -1893.625527

G_{sp} = -1895.326986

N_{imag} = 0

SP = -1895.979091

C	3.433659000000	-2.617368000000	1.556158000000
C	2.037893000000	-2.565084000000	1.472056000000
C	1.460248000000	-2.814530000000	0.217779000000
C	2.213440000000	-3.109907000000	-0.932131000000
C	3.602775000000	-3.153479000000	-0.793141000000
C	4.207154000000	-2.907251000000	0.437906000000
C	1.213954000000	-2.264873000000	2.713764000000
C	1.549751000000	-3.298420000000	-2.287341000000
C	1.408761000000	-1.945295000000	-2.997588000000
C	2.282342000000	-4.302786000000	-3.177646000000
C	1.338515000000	-3.404514000000	3.730998000000
C	1.601568000000	-0.919092000000	3.333150000000
N	0.029778000000	-2.730949000000	0.098212000000
C	-0.840954000000	-3.805994000000	0.122212000000
C	-2.094357000000	-3.288006000000	0.033375000000
N	-1.943265000000	-1.914277000000	-0.025860000000
C	-0.639751000000	-1.560899000000	0.011381000000
C	-3.001755000000	-0.949846000000	-0.142998000000
C	-3.307476000000	-0.450534000000	-1.418465000000
C	-4.287272000000	0.545003000000	-1.497713000000
C	-4.923258000000	1.015161000000	-0.353545000000
C	-4.606720000000	0.488129000000	0.896957000000
C	-3.640324000000	-0.512639000000	1.030676000000
C	-3.270986000000	-1.072763000000	2.396305000000
C	-2.220770000000	-0.193152000000	3.085510000000
C	-4.491064000000	-1.266618000000	3.299058000000
C	-2.628285000000	-0.955196000000	-2.682138000000
C	-3.651412000000	-1.598010000000	-3.624954000000
C	-1.847435000000	0.158448000000	-3.386467000000
Au	0.132243000000	0.359233000000	-0.026128000000
C	0.992155000000	2.221087000000	-0.054506000000
Sb	3.046362000000	2.355308000000	-0.181685000000
H	3.919899000000	-2.425566000000	2.514573000000
H	4.224833000000	-3.374199000000	-1.660982000000
H	5.294225000000	-2.939193000000	0.523351000000
H	0.155707000000	-2.197913000000	2.420197000000
H	0.535870000000	-3.691031000000	-2.114462000000
H	0.903952000000	-2.070261000000	-3.967368000000
H	0.831344000000	-1.228417000000	-2.394872000000
H	2.401079000000	-1.504569000000	-3.178357000000
H	1.687195000000	-4.504587000000	-4.079399000000
H	2.456096000000	-5.255566000000	-2.657312000000
H	3.254295000000	-3.911465000000	-3.512234000000
H	0.709442000000	-3.203043000000	4.610437000000
H	1.026743000000	-4.364598000000	3.294564000000
H	2.378106000000	-3.510672000000	4.076515000000
H	0.956087000000	-0.698749000000	4.196023000000
H	1.498254000000	-0.100509000000	2.604455000000
H	2.643195000000	-0.928197000000	3.687977000000
H	-0.489547000000	-4.830618000000	0.196497000000
H	-3.070151000000	-3.763362000000	0.010658000000
H	-4.548451000000	0.963562000000	-2.471898000000
H	-5.678015000000	1.798854000000	-0.435455000000
H	-5.120082000000	0.863032000000	1.783440000000
H	-2.819393000000	-2.064042000000	2.239933000000
H	-1.935410000000	-0.627974000000	4.055345000000
H	-1.312079000000	-0.089872000000	2.472498000000
H	-2.622701000000	0.815784000000	3.266213000000
H	-4.196464000000	-1.795571000000	4.216483000000

H	-5.275653000000	-1.851866000000	2.798664000000
H	-4.924213000000	-0.302959000000	3.604872000000
H	-1.904534000000	-1.734060000000	-2.398205000000
H	-3.146680000000	-2.013705000000	-4.509001000000
H	-4.199906000000	-2.409544000000	-3.125384000000
H	-4.386337000000	-0.857598000000	-3.975355000000
H	-1.372394000000	-0.229941000000	-4.299201000000
H	-1.058511000000	0.565979000000	-2.736358000000
H	-2.511371000000	0.987375000000	-3.676893000000
N	2.509999000000	4.381216000000	-0.111734000000
N	1.295915000000	4.584657000000	-0.030747000000
N	0.456312000000	3.437702000000	0.001035000000
C	-1.005493000000	3.747899000000	0.104732000000
C	-1.734321000000	3.155647000000	-1.104064000000
H	-1.711187000000	2.056952000000	-1.090055000000
H	-2.788993000000	3.467930000000	-1.085748000000
H	-1.277100000000	3.507967000000	-2.040601000000
C	-1.553253000000	3.149801000000	1.402595000000
H	-2.617364000000	3.409321000000	1.506816000000
H	-1.468570000000	2.054360000000	1.401864000000
H	-1.006045000000	3.546049000000	2.270626000000
C	-1.229503000000	5.262441000000	0.122760000000
H	-0.853991000000	5.735470000000	-0.792324000000
H	-2.312078000000	5.439582000000	0.199281000000
H	-0.728893000000	5.734662000000	0.975878000000

1-Bi
***E* =** -1546.252287
***H* =** -1546.215813
***G* =** -1546.325600
G_{sp} = -1547.662361
N_{imag} = 0
***SP* =** -1548.180166

C	-1.207102000000	4.263347000000	-1.381750000000
C	-1.651171000000	2.936932000000	-1.340758000000
C	-2.044160000000	2.429329000000	-0.093494000000
C	-2.010678000000	3.186022000000	1.091390000000
C	-1.562302000000	4.505437000000	0.995362000000
C	-1.162914000000	5.038448000000	-0.228646000000
C	-1.703435000000	2.113668000000	-2.617391000000
C	-2.379507000000	2.572547000000	2.433365000000
C	-1.144985000000	1.927880000000	3.078232000000
C	-3.028141000000	3.573214000000	3.390403000000
C	-2.727175000000	2.697197000000	-3.597146000000
C	-0.318783000000	1.994240000000	-3.261039000000
N	-2.493658000000	1.066284000000	-0.022229000000
C	-3.820700000000	0.674876000000	-0.019074000000
C	-3.819109000000	-0.682547000000	0.020856000000
N	-2.491177000000	-1.070859000000	0.023112000000
C	-1.663908000000	-0.001330000000	0.000192000000
C	-2.038473000000	-2.432848000000	0.093875000000
C	-1.643750000000	-2.939819000000	1.340857000000
C	-1.196471000000	-4.265164000000	1.381326000000
C	-1.150830000000	-5.039837000000	0.227979000000
C	-1.551924000000	-4.507452000000	-0.995740000000
C	-2.003601000000	-3.189124000000	-1.091224000000
C	-2.374382000000	-2.576201000000	-2.432898000000
C	-1.141397000000	-1.929339000000	-3.078478000000
C	-3.021922000000	-3.577971000000	-3.389543000000
C	-1.697528000000	-2.116931000000	2.617672000000

C	-2.719821000000	-2.702980000000	3.597444000000
C	-0.313051000000	-1.994458000000	3.261091000000
Au	0.423041000000	0.000440000000	-0.000040000000
C	2.407870000000	0.001999000000	-0.000143000000
Bi	4.353483000000	0.003612000000	-0.000282000000
H	-0.890270000000	4.692525000000	-2.334354000000
H	-1.517267000000	5.126108000000	1.890743000000
H	-0.811256000000	6.069869000000	-0.280950000000
H	-2.033797000000	1.096364000000	-2.360311000000
H	-3.113205000000	1.773163000000	2.247918000000
H	-1.416169000000	1.447395000000	4.030503000000
H	-0.690912000000	1.169957000000	2.422173000000
H	-0.379715000000	2.692214000000	3.282110000000
H	-3.397800000000	3.049066000000	4.283042000000
H	-3.874077000000	4.095259000000	2.920601000000
H	-2.306311000000	4.328553000000	3.734365000000
H	-2.790486000000	2.073242000000	-4.500668000000
H	-3.727476000000	2.751874000000	-3.143403000000
H	-2.440089000000	3.713095000000	-3.908577000000
H	-0.367946000000	1.348214000000	-4.149927000000
H	0.410018000000	1.562205000000	-2.558544000000
H	0.056501000000	2.978185000000	-3.581185000000
H	-4.633565000000	1.394250000000	-0.041281000000
H	-4.630262000000	-1.403842000000	0.043645000000
H	-0.878260000000	-4.693849000000	2.333692000000
H	-0.796642000000	-6.070414000000	0.279859000000
H	-1.505674000000	-5.127744000000	-1.891328000000
H	-3.109351000000	-1.778111000000	-2.246934000000
H	-1.414025000000	-1.449147000000	-4.030485000000
H	-0.688204000000	-1.170762000000	-2.422553000000
H	-0.374947000000	-2.692323000000	-3.282986000000
H	-3.393008000000	-3.054461000000	-4.281966000000
H	-3.866695000000	-4.101416000000	-2.919208000000
H	-2.299046000000	-4.332119000000	-3.733922000000
H	-2.030200000000	-1.100315000000	2.360874000000
H	-2.784342000000	-2.079480000000	4.501194000000
H	-3.720077000000	-2.759676000000	3.143845000000
H	-2.430459000000	-3.718366000000	3.908435000000
H	-0.363554000000	-1.348661000000	4.150070000000
H	0.414616000000	-1.560627000000	2.558521000000
H	0.064574000000	-2.977561000000	3.581071000000

TS-Bi

***E* =** -1867.761342

***H* =** -1867.715819

***G* =** -1867.845785

G_{sp} = -1869.551758

N_{imag} = 1, -242 cm^{-1}

***SP* =** -1870.197079

C	-2.036365000000	4.305052000000	-1.370172000000
C	-2.555565000000	3.022714000000	-1.164459000000
C	-2.566231000000	2.536164000000	0.151457000000
C	-2.079719000000	3.269606000000	1.246742000000
C	-1.570318000000	4.544980000000	0.987747000000
C	-1.548994000000	5.058007000000	-0.306778000000
C	-3.057364000000	2.203858000000	-2.342139000000
C	-2.042187000000	2.687884000000	2.650989000000
C	-0.633041000000	2.179618000000	2.981125000000
C	-2.529528000000	3.683294000000	3.706431000000
C	-4.207065000000	2.914389000000	-3.062901000000
C	-1.910672000000	1.876837000000	-3.304318000000

N	-3.071887000000	1.213274000000	0.387728000000
C	-4.364903000000	0.920548000000	0.783831000000
C	-4.427484000000	-0.429718000000	0.912472000000
N	-3.174506000000	-0.912847000000	0.576423000000
C	-2.326809000000	0.092662000000	0.256555000000
C	-2.794608000000	-2.297425000000	0.628303000000
C	-2.074330000000	-2.751440000000	1.743575000000
C	-1.722659000000	-4.105945000000	1.770782000000
C	-2.076789000000	-4.958521000000	0.731912000000
C	-2.788258000000	-4.475960000000	-0.365011000000
C	-3.160100000000	-3.132030000000	-0.443363000000
C	-3.865947000000	-2.574896000000	-1.670117000000
C	-2.836540000000	-2.010843000000	-2.659282000000
C	-4.771397000000	-3.595934000000	-2.358880000000
C	-1.691119000000	-1.846428000000	2.903472000000
C	-2.462158000000	-2.238110000000	4.168840000000
C	-0.178597000000	-1.852223000000	3.143679000000
Au	-0.308978000000	-0.037045000000	-0.223886000000
C	1.640760000000	-0.151089000000	-0.618769000000
Bi	3.336333000000	-0.377781000000	-1.604143000000
H	-2.005967000000	4.715004000000	-2.381747000000
H	-1.173704000000	5.142527000000	1.810107000000
H	-1.141889000000	6.053797000000	-0.488427000000
H	-3.446440000000	1.249438000000	-1.956821000000
H	-2.721932000000	1.822897000000	2.678378000000
H	-0.614585000000	1.729430000000	3.985383000000
H	-0.289513000000	1.426542000000	2.255588000000
H	0.089220000000	3.010400000000	2.964360000000
H	-2.585116000000	3.192079000000	4.688492000000
H	-3.525855000000	4.077749000000	3.459999000000
H	-1.840054000000	4.534673000000	3.806242000000
H	-4.589289000000	2.287471000000	-3.881661000000
H	-5.037807000000	3.129832000000	-2.375362000000
H	-3.872691000000	3.867455000000	-3.499969000000
H	-2.271045000000	1.239934000000	-4.125678000000
H	-1.096706000000	1.348818000000	-2.784691000000
H	-1.494307000000	2.795583000000	-3.745253000000
H	-5.109016000000	1.695410000000	0.940060000000
H	-5.238811000000	-1.088864000000	1.205955000000
H	-1.161600000000	-4.495435000000	2.622434000000
H	-1.792807000000	-6.011160000000	0.772451000000
H	-3.051827000000	-5.157108000000	-1.174446000000
H	-4.503112000000	-1.739636000000	-1.341079000000
H	-3.343430000000	-1.574401000000	-3.533204000000
H	-2.207642000000	-1.235479000000	-2.196936000000
H	-2.170262000000	-2.813225000000	-3.010884000000
H	-5.358845000000	-3.101588000000	-3.145414000000
H	-5.468676000000	-4.065600000000	-1.650210000000
H	-4.186035000000	-4.391231000000	-2.843642000000
H	-1.975090000000	-0.815315000000	2.646792000000
H	-2.212655000000	-1.556474000000	4.995317000000
H	-3.548774000000	-2.198040000000	4.003208000000
H	-2.205124000000	-3.260824000000	4.484865000000
H	0.080770000000	-1.140976000000	3.941863000000
H	0.370959000000	-1.557966000000	2.236914000000
H	0.171631000000	-2.848202000000	3.455432000000
N	4.528774000000	0.095375000000	0.774018000000
N	3.557999000000	0.264576000000	1.529706000000
N	2.433977000000	0.326735000000	1.730967000000
C	5.896931000000	0.161969000000	1.326635000000
C	6.827908000000	-0.087951000000	0.144144000000

H	6.631735000000	-1.075698000000	-0.297339000000
H	6.676307000000	0.675705000000	-0.632285000000
H	7.875195000000	-0.051415000000	0.475167000000
C	6.149347000000	1.549649000000	1.920433000000
H	5.996007000000	2.324724000000	1.156329000000
H	5.460097000000	1.742867000000	2.756209000000
H	7.179567000000	1.624485000000	2.298034000000
C	6.080972000000	-0.923647000000	2.389440000000
H	5.390298000000	-0.761212000000	3.230601000000
H	5.879750000000	-1.915343000000	1.960189000000
H	7.109212000000	-0.909488000000	2.779361000000

2-Bi

***E* =** -1867.863324

***H* =** -1867.818836

***G* =** -1867.946121

G_{sp} = -1869.647684

N_{imag} = 0

***SP* =** -1870.297281

C	-1.461339000000	4.469365000000	-0.692747000000
C	-2.085042000000	3.225147000000	-0.828927000000
C	-2.386610000000	2.523703000000	0.348751000000
C	-2.086918000000	3.011300000000	1.631172000000
C	-1.463064000000	4.259667000000	1.713456000000
C	-1.153989000000	4.981976000000	0.564219000000
C	-2.372290000000	2.658070000000	-2.209368000000
C	-2.368822000000	2.206255000000	2.889363000000
C	-1.065271000000	1.643469000000	3.468149000000
C	-3.133071000000	3.024662000000	3.933239000000
C	-3.202291000000	3.624688000000	-3.058590000000
C	-1.064767000000	2.279371000000	-2.914144000000
N	-3.000012000000	1.230284000000	0.238069000000
C	-4.360034000000	0.987637000000	0.317212000000
C	-4.513782000000	-0.356728000000	0.199079000000
N	-3.243914000000	-0.883665000000	0.040510000000
C	-2.301249000000	0.086232000000	0.071382000000
C	-2.949192000000	-2.284199000000	-0.101139000000
C	-2.449586000000	-2.983401000000	1.008274000000
C	-2.183525000000	-4.347193000000	0.837717000000
C	-2.405774000000	-4.973949000000	-0.382289000000
C	-2.890977000000	-4.249300000000	-1.469327000000
C	-3.167189000000	-2.885277000000	-1.354817000000
C	-3.602773000000	-2.060859000000	-2.556930000000
C	-2.381070000000	-1.386765000000	-3.197515000000
C	-4.377504000000	-2.869236000000	-3.597064000000
C	-2.223164000000	-2.332087000000	2.363020000000
C	-3.298583000000	-2.787551000000	3.356443000000
C	-0.814590000000	-2.602999000000	2.900934000000
Au	-0.232360000000	-0.177929000000	0.087882000000
C	1.780173000000	-0.465172000000	0.201232000000
Bi	3.262041000000	0.708240000000	-0.809898000000
H	-1.204870000000	5.041763000000	-1.586394000000
H	-1.205084000000	4.667305000000	2.692642000000
H	-0.662319000000	5.952201000000	0.649187000000
H	-2.960449000000	1.736126000000	-2.086221000000
H	-3.004104000000	1.351536000000	2.613382000000
H	-1.274818000000	1.026514000000	4.354798000000
H	-0.534656000000	1.022967000000	2.729429000000
H	-0.390873000000	2.459211000000	3.770980000000
H	-3.386218000000	2.393023000000	4.796853000000

H	-4.065750000000	3.433641000000	3.518690000000
H	-2.528744000000	3.865150000000	4.305721000000
H	-3.449613000000	3.163345000000	-4.025611000000
H	-4.141102000000	3.895293000000	-2.554442000000
H	-2.648156000000	4.552351000000	-3.266107000000
H	-1.272520000000	1.831069000000	-3.896902000000
H	-0.491177000000	1.554333000000	-2.316208000000
H	-0.435294000000	3.169092000000	-3.070898000000
H	-5.081893000000	1.786844000000	0.453865000000
H	-5.400543000000	-0.983226000000	0.208697000000
H	-1.794279000000	-4.924151000000	1.678306000000
H	-2.191942000000	-6.038044000000	-0.492836000000
H	-3.047617000000	-4.754256000000	-2.422624000000
H	-4.271404000000	-1.264102000000	-2.196661000000
H	-2.690564000000	-0.750444000000	-4.040498000000
H	-1.831908000000	-0.765754000000	-2.474133000000
H	-1.684598000000	-2.149732000000	-3.577172000000
H	-4.781250000000	-2.196055000000	-4.366389000000
H	-5.215538000000	-3.418422000000	-3.144332000000
H	-3.727859000000	-3.594226000000	-4.109334000000
H	-2.321333000000	-1.243554000000	2.243283000000
H	-3.157804000000	-2.291483000000	4.328156000000
H	-4.308522000000	-2.550887000000	2.990071000000
H	-3.243570000000	-3.874983000000	3.519400000000
H	-0.659422000000	-2.041282000000	3.834064000000
H	-0.031358000000	-2.302772000000	2.186928000000
H	-0.675075000000	-3.669752000000	3.133681000000
N	4.343547000000	-0.826722000000	0.352676000000
N	3.591447000000	-1.617959000000	1.025935000000
N	2.290207000000	-1.449785000000	0.961401000000
C	5.797573000000	-1.089726000000	0.479469000000
C	6.546675000000	-0.066075000000	-0.368924000000
H	6.262134000000	-0.149571000000	-1.429051000000
H	6.340248000000	0.958230000000	-0.022692000000
H	7.629289000000	-0.240163000000	-0.292172000000
C	6.199744000000	-0.946713000000	1.949512000000
H	5.969486000000	0.066735000000	2.309699000000
H	5.646875000000	-1.670034000000	2.562124000000
H	7.278582000000	-1.125345000000	2.065245000000
C	6.088936000000	-2.504486000000	-0.027762000000
H	5.534725000000	-3.239550000000	0.569734000000
H	5.780022000000	-2.602474000000	-1.078875000000
H	7.165217000000	-2.718027000000	0.044840000000

TS-iso-Bi
E **=** -1867.755943
H **=** -1867.710814
G **=** -1867.838948
G_{sp} **=** -1869.545816
N_{imag} **=** 1, -303 cm^{-1}
SP **=** -1870.191823

C	0.857614000000	4.829352000000	0.409551000000
C	1.548164000000	3.653128000000	0.713849000000
C	1.894684000000	2.813954000000	-0.358093000000
C	1.569120000000	3.098908000000	-1.692889000000
C	0.877036000000	4.287897000000	-1.944095000000
C	0.526468000000	5.144773000000	-0.905754000000
C	1.844307000000	3.278132000000	2.157070000000
C	1.919304000000	2.163973000000	-2.838724000000
C	0.650964000000	1.617946000000	-3.503219000000

C	2.831511000000	2.853212000000	-3.858236000000
C	2.435929000000	4.443949000000	2.952425000000
C	0.579906000000	2.731416000000	2.832005000000
N	2.587664000000	1.589338000000	-0.069024000000
C	3.955043000000	1.476889000000	0.104283000000
C	4.199066000000	0.167618000000	0.368841000000
N	2.972690000000	-0.474145000000	0.333542000000
C	1.969816000000	0.394949000000	0.069947000000
C	2.795445000000	-1.878664000000	0.579368000000
C	3.101071000000	-2.778722000000	-0.458472000000
C	2.976835000000	-4.142099000000	-0.183742000000
C	2.548352000000	-4.583506000000	1.066895000000
C	2.224294000000	-3.668581000000	2.061237000000
C	2.340525000000	-2.290820000000	1.840594000000
C	1.982395000000	-1.315928000000	2.949791000000
C	0.533144000000	-1.509657000000	3.407886000000
C	2.963544000000	-1.439137000000	4.120246000000
C	3.456141000000	-2.281931000000	-1.851164000000
C	4.375267000000	-3.232092000000	-2.618023000000
C	2.168198000000	-2.014363000000	-2.643139000000
Au	-0.080258000000	0.066011000000	0.003025000000
C	-2.072039000000	-0.119405000000	-0.003062000000
Bi	-3.829341000000	0.793325000000	0.177343000000
H	0.563528000000	5.503831000000	1.215370000000
H	0.601604000000	4.540823000000	-2.969910000000
H	-0.018108000000	6.065058000000	-1.121898000000
H	2.591956000000	2.470978000000	2.153310000000
H	2.471267000000	1.305663000000	-2.426965000000
H	0.912890000000	0.897109000000	-4.291942000000
H	0.004131000000	1.113513000000	-2.768897000000
H	0.069176000000	2.429630000000	-3.966180000000
H	3.116356000000	2.148501000000	-4.653092000000
H	3.749524000000	3.228736000000	-3.383466000000
H	2.322369000000	3.705268000000	-4.333530000000
H	2.720490000000	4.106479000000	3.959362000000
H	3.328910000000	4.855339000000	2.460163000000
H	1.707343000000	5.259084000000	3.074274000000
H	0.801007000000	2.416143000000	3.862947000000
H	0.171365000000	1.869727000000	2.281614000000
H	-0.202512000000	3.504882000000	2.869655000000
H	4.618149000000	2.333157000000	0.028834000000
H	5.121720000000	-0.366949000000	0.573050000000
H	3.208675000000	-4.872562000000	-0.959424000000
H	2.455811000000	-5.653127000000	1.261578000000
H	1.875208000000	-4.026199000000	3.031984000000
H	2.063870000000	-0.294026000000	2.552009000000
H	0.267777000000	-0.747552000000	4.155284000000
H	-0.168261000000	-1.420253000000	2.563590000000
H	0.390467000000	-2.497401000000	3.872242000000
H	2.719032000000	-0.706140000000	4.902945000000
H	3.998784000000	-1.263641000000	3.793014000000
H	2.915640000000	-2.442457000000	4.570541000000
H	3.986761000000	-1.323318000000	-1.746227000000
H	4.701615000000	-2.759065000000	-3.554864000000
H	5.268909000000	-3.493404000000	-2.033294000000
H	3.857190000000	-4.163802000000	-2.889350000000
H	2.403935000000	-1.615756000000	-3.641476000000
H	1.515431000000	-1.293741000000	-2.126269000000
H	1.599904000000	-2.949220000000	-2.769515000000
N	-4.855222000000	-1.761929000000	0.010050000000
N	-3.852050000000	-2.329816000000	-0.065894000000

N	-2.625720000000	-2.383365000000	0.006276000000
C	-1.898908000000	-3.342494000000	-0.866578000000
C	-1.745031000000	-2.734416000000	-2.262460000000
H	-1.176367000000	-1.795389000000	-2.206898000000
H	-1.219652000000	-3.438063000000	-2.926048000000
H	-2.731468000000	-2.514040000000	-2.698353000000
C	-0.537638000000	-3.585844000000	-0.224822000000
H	0.037723000000	-4.303106000000	-0.828286000000
H	0.041269000000	-2.652185000000	-0.152658000000
H	-0.658782000000	-3.995658000000	0.788493000000
C	-2.674670000000	-4.660353000000	-0.939415000000
H	-3.656018000000	-4.513752000000	-1.413418000000
H	-2.108544000000	-5.392699000000	-1.532684000000
H	-2.831065000000	-5.070273000000	0.068669000000
